# A mechanistic systems biology model of brain microvascular endothelial cell signaling reveals dynamic pathway-based therapeutic targets for brain ischemia

**DOI:** 10.1016/j.redox.2024.103415

**Published:** 2024-11-05

**Authors:** Geli Li, Yuchen Ma, Sujie Zhang, Wen Lin, Xinyi Yao, Yating Zhou, Yanyong Zhao, Qi Rao, Yuchen Qu, Yuan Gao, Lianmin Chen, Yu Zhang, Feng Han, Meiling Sun, Chen Zhao

**Affiliations:** a School of Pharmacy, Nanjing Medical University, 210000, Nanjing, China; b Gusu School, Nanjing Medical University, 215000, Suzhou, China; c The First Affiliated Hospital of Nanjing Medical University, 210000, Nanjing, China; d QSPMed Technologies, 210000, Nanjing, China; e Department of Biomedical Engineering, School of Medicine, Johns Hopkins University, 21205, Baltimore, USA; f Key Laboratory of Cardiovascular & Cerebrovascular Medicine, Drug Target and Drug Discovery Center, School of Pharmacy, Nanjing Medical University, 210000, Nanjing, China; g School of Basic Medical Sciences, Nanjing Medical University, 210000, Nanjing, China

**Keywords:** Systems biology, Brain microvascular endothelial cell, Ischemic stroke, Oxidative stress, Model-informed drug target assessment

## Abstract

Ischemic stroke is a significant threat to human health. Currently, there is a lack of effective treatments for stroke, and progress in new neuron-centered drug target development is relatively slow. On the other hand, studies have demonstrated that brain microvascular endothelial cells (BMECs) are crucial components of the neurovascular unit and play pivotal roles in ischemic stroke progression. To better understand the complex multifaceted roles of BMECs in the regulation of ischemic stroke pathophysiology and facilitate BMEC-based drug target discovery, we utilized a transcriptomics-informed systems biology modeling approach and constructed a mechanism-based computational multipathway model to systematically investigate BMEC function and its modulatory potential. Extensive multilevel data regarding complex BMEC pathway signal transduction and biomarker expression under various pathophysiological conditions were used for quantitative model calibration and validation, and we generated dynamic BMEC phenotype maps in response to various stroke-related stimuli to identify potential determinants of BMEC fate under stress conditions. Through high-throughput model sensitivity analyses and virtual target perturbations in model-based single cells, our model predicted that targeting succinate could effectively reverse the detrimental cell phenotype of BMECs under oxygen and glucose deprivation/reoxygenation, a condition that mimics stroke pathogenesis, and we experimentally validated the utility of this new target in terms of regulating inflammatory factor production, free radical generation and tight junction protection *in vitro* and *in vivo*. Our work is the first that complementarily couples transcriptomic analysis with mechanistic systems-level pathway modeling in the study of BMEC function and endothelium-based therapeutic targets in ischemic stroke.

## 1 Introduction

Epidemiological data have revealed a concerning increase in the global annual incidence of ischemic stroke, which poses a significant threat to human health [1]. Through extensive research on stroke pathophysiology, researchers have discovered that brain microvascular endothelial cells (BMECs), which differ from conventional endothelial cells due to their unique structure and function, are crucial components of the blood-brain barrier (BBB) [2]. Studies have shown that BMECs form tightly sealed barrier structures through interconnections facilitated by tight junction proteins such as zona occludens 1 (ZO-1) and Claudin5, which can effectively restrict the undesired entry of polar and toxic substances across the BBB [3,4]. BMECs also express and secrete a variety of chemokines and cytokines to recruit immune cells and thereby mediate inflammatory processes within the tissue microenvironment [5,6]. By nature, BMECs play a pivotal role in maintaining the permeability and integrity of the BBB. Once ischemic stroke occurs, external stimuli such as cytokines, chemokines and reactive oxygen species can damage the tight junction proteins of BMECs, resulting in the loss of tight junction functionality [7]. The activation of apoptotic pathways within these cells leads to increased cell death, further compromise the normal functioning of the BBB and permitting the infiltration of toxic substances into the central nervous system (CNS), thus exacerbating the condition [[8], [9], [10]]. It is now evident that minimizing the extent of BMEC damage is of significant importance for maintaining the fundamental integrity of the BBB following ischemic stroke, and this provides new therapeutic opportunities for drug target discovery [11,12]. Currently, there is a lack of safe and effective therapeutic interventions for the recovery of brain tissue function following ischemic stroke. Drugs developed with a sole focus on neuronal protection have encountered significant translational challenges in clinical trials [13]. Therefore, investigating new drug targets and therapeutic mechanisms related to the function and protection of cerebral microvascular endothelial cells holds crucial translational significance for ischemic stroke drug discovery.

During ischemic stroke, BMECs themselves are exposed to and delicately regulated by a complex microenvironment within the brain that is dynamically comprised of a spectrum of inflammatory factors, growth factors, metabolites and other types of cells. Moreover, changes in internal cellular metabolism and oxidative stress pathways also have profound impacts on the fate of BMECs [[14], [15], [16]]. For example, during cerebral ischemia-reperfusion, a substantial amount of inflammatory factors produced by the microenvironment can interact with surface receptors on BMECs, such as TNFR (tumor necrosis factor receptor) and TLR4 (toll like receptor 4), thereby triggering downstream activation involving the NFкB (nuclear factor kappa B) axis [17,18]. Consequently, this cascade promotes the secretion of pro-inflammatory cytokines such as IL-6 (interleukin 6) and IL-1β (interleukin 1 beta) by BMECs which can potentially result in inflammatory storms that occur in a vicious cycle [19]. Concurrently, the levels of O_2_^−^ (superoxide) and ONOO^−^ (peroxynitrite), along with other free radicals, rapidly increase within BMECs and result in oxidative stress-induced damage and activation of the caspase pathways to drive cellular apoptosis [[20], [21], [22], [23]]. In response to this, intracellular signaling pathways related to BMEC viability can also be activated to counteract and repair damage. For example, binding of VEGF (Vascular endothelial growth factor) and FGF (Fibroblast growth factor) with their respective receptors on BMECs strongly activates the PLCγ (phospholipase C gamma)/MAPK (mitogen-activated protein kinases) and classic PI3K/AKT pathways which promote cell proliferation and survival [24]. Another key factor involved in the pathophysiology of ischemic stroke is hypoxia, which is the master regulator of intracellular oxygen sensing and adaptation and can induce the synthesis of pro-growth and vessel-stabilizing factors such as Ang2 (angiopoietin 2), SEMA3G (semaphorin 3G), and BDNF (brain derived neurotrophic factor) [[25], [26], [27]]. In addition, the glucose metabolism pathway is crucial for cell survival. Following ischemic stroke, the deprivation of glucose and oxygen leads to a decrease in the intracellular ratio of ATP to AMP, activating multiple signaling pathways and accelerating cellular apoptosis [28,29]. Given the highly complex network of pathway interactions within BMECs, understanding the driving principles of this network in terms of how it dynamically responds to various simultaneous signals during stroke is key to the identification of new endothelium-based therapeutic targets [30]. Therefore, a systems biology modeling approach that integrates pathway data will greatly facilitate our quantitative and mechanistic understanding of this intricate BMEC cell response in stroke, where the microenvironment is complex and multifactorial by nature.

To our knowledge, only one computational model has been developed so far for the quantitative analysis of BMEC signaling [31]. This logic-based theoretical model by Gorick et al. focuses primarily on the effects of external growth signals on the regulation of tight junction proteins in cerebral pathologies; however given its scope, mechanistic details regarding intracellular stress sensing, metabolism, oxidative damage and inflammatory signaling were not included and experimental validation under stroke-related conditions was not carried out. Therefore, here we constructed a transcriptomics-informed, mechanistically advanced data-driven computational model based on mass-action and Hill-type kinetics and used it to integratively characterize BMEC physiology in stroke through combined simulation and experimental investigations. Our model is the first to include mechanistic modules of BMEC growth factor signaling, inflammatory signaling, oxygen sensing, oxidative stress and free radical generation, apoptosis, glucose metabolism, tight junction proteins and cytokine biomarker production, and we comprehensively incorporated numerous sets of quantitative experimental data (consisting of over 300 data points) into the model calibration and validation process. Using this model, we systematically explored the temporal changes in crucial signaling pathways in BMECs under classic OGD/R conditions (oxygen and glucose deprivation/reoxygenation, the most-commonly used *in vitro* condition for studying ischemic stroke) and quantified the impact of different ischemic/reperfusion durations on the fate of BMECs. We generated a population of model-based “virtual” BMECs and investigated the changes in diverse cell phenotypes under OGD/R conditions upon different target protein perturbations. Through model sensitivity analyses and *in silico* target efficacy evaluation, we discovered that succinate is an essential node that can significantly alter BMEC functional response upon stress, and we experimentally validated this prediction and provided *in vitro* and *in vivo* biological evidence regarding the potential therapeutic value of modulating this axis to improve BMEC function and alleviate injury in brain ischemia. Our mechanistic model can serve as a valuable proof-of-concept platform to advance our understanding of the pathway network-based multifaceted function of BMECs in ischemic stroke and other cerebral disorders, while providing high-throughput application scenarios for translational research in terms of comprehensive cellular simulation and drug target evaluation.

## 2 Results

### 2.1 Transcriptomic analysis reveals key pathways involved in brain microvascular endothelial cell function following experimental ischemic stroke and mechanistically informs systems biology model formulation

**Fig. 1:**
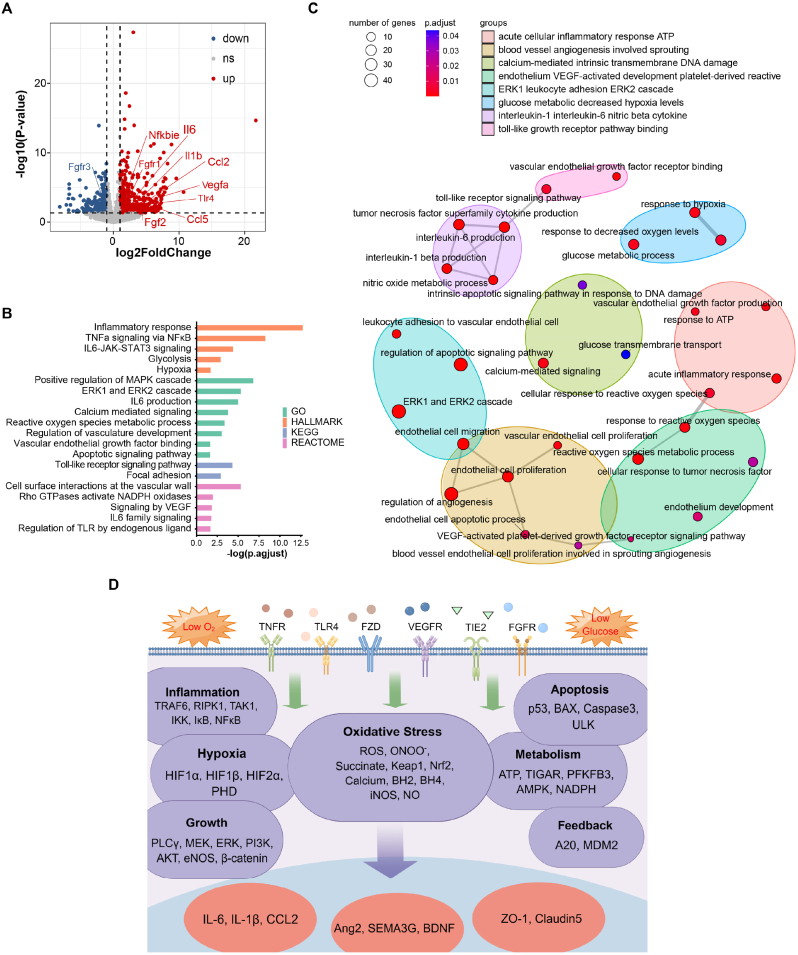
Bioinformatics analyses of differentially regulated BMEC transcriptome from experimental stroke models critically informed mechanism-based formulation of the BMEC computational model. (A) The volcano plot was used to visualize the differentially expressed genes (DEGs) in BMECs isolated from ischemic and control hemispheres in mice undergoing tMCAO procedures (raw dataset from GSE163752). The P value < 0.05 and |log(2)Fold Change| > 1 were considered as thresholds. Red dots represent upregulated genes while blue dots represent downregulated genes. Gray dots represent no significant changes. (B) GSEA analysis of DEGs using hallmark gene sets, curated gene sets and gene ontology (GO) sets to visualize the most essential pathways associated with experimental stroke pathophysiology in BMECs. Significant terms were identified with P.adjust <0.05. (C) The top 30 GO pathway results were mapped into a pathway interaction network and visualized accordingly. Nodes represent enriched GO terms with color intensities reflecting statistical significance (node size reflects number of genes involved). Significant terms were identified with P.adjust <0.05. (D) Structurally and mechanistically informed by the bioinformatics analysis results, a detailed computational model comprising multiple pathway modules of BMEC signaling and function (including inflammation, cell survival, hypoxia regulation, cell metabolism, and oxidative stress) was constructed and encoded in ordinary differential equations. See Methods and Supplementary Table S1 for details.

To mechanistically identify the pivotal molecular determinants of ischemic stroke-induced BMEC injury and select pathways to be included in the subsequent model-building step, we conducted transcriptomic analysis of Gene Expression Omnibus (GEO) dataset GSE163752, in which BMECs were isolated from healthy-appearing contralateral hemisphere and stroke-affected ipsilateral hemisphere of mice after transient middle cerebral artery occlusion (tMCAO, a widely used experimental stroke procedure in rodents). Differential gene expression analysis indicated that a large number of genes were significantly up-/downregulated in BMECs following tMCAO, among which many were notably related to biological processes such as inflammation and growth factor signaling (Fig. 1A). Then, we performed GSEA with differentially expressed genes to further investigate the functional changes that occur in BMECs after tMCAO. The results revealed that pathways associated with apoptosis (e.g. “Apoptotic signaling pathway”), inflammation (e.g. “Inflammatory response”, “TNFα signaling via NFκB”, “Toll-like receptor signaling pathway”), energy metabolism (e.g. “Glycolysis”, “Hypoxia”), oxidative stress (e.g. “Reactive oxygen species metabolic process”), cell survival (e.g. “ERK1 and ERK2 cascade”, “Vascular endothelial growth factor binding”, “Signaling by VEGF”) and cellular function (e.g. “Regulation of vasculature development”, “Focal adhesion”, “Cell surface interactions at the vascular wall”) were significantly enriched (Fig. 1B). Furthermore, the top 30 candidates derived from the pathway analysis of BMECs following tMCAO were then mapped to an interaction network (Fig. 1C), which again suggested that the pathways generally related to inflammatory responses, energy metabolism and cell survival in BMECs changed markedly after experimental stroke in mice. These transcriptomics-based results provided key insights for subsequent structural formulation of the BMEC computational systems biology model (Fig. 1D).

Based on the above gene and pathway enrichment results and literature survey, we mechanistically formulated a computational systems biology model of BMEC physiology. The entire model is divided into several mechanistic modules as informed by the aforementioned pathway analysis: inflammatory pathway module, cell survival module, hypoxia regulation module, cell metabolism module, and oxidative stress module. To better depict the pathophysiological changes of BMECs under OGD/R conditions, the model centers on the oxidative stress module which interacts with other modules, and model formulation is based on mass action and hill-type kinetics to describe the biochemical reactions of cell signaling and transduction (Fig. 1D). The inflammatory pathway module describes the binding of TNFR and TLR4 receptors with inflammatory ligands which induces the activation of downstream proteins such as TRAF6 (TNF receptor associated factor 6), RIPK1 (receptor interacting protein kinase 1), TAK1 (transforming growth factor β-activated kinase 1) and IKK (inhibitor of kappa B kinase). This leads to the subsequent nuclear translocation of NFкB and promotes the secretion of the inflammatory cytokines IL-6 and IL-1β, and the chemokine CCL2 (C–C motif chemokine ligand 2) [19]. Additionally, the model incorporates the negative feedback regulation of A20 (TNF alpha induced protein 3) on TRAF6 and RIPK1 ubiquitination [32,33]. The cell survival module describes the phosphorylation of VEGFR (Vascular endothelial growth factor receptor) at Y1175 and Y951 upon binding with soluble VEGF, with Y1175 phosphorylation activating the PLCγ/MEK/ERK pathway and Y951 phosphorylation activating the PI3K/AKT pathway [24]. It also includes Tie signaling: when Ang1 (angiopoietin 1) binds to Tie2, phosphorylated Tie2 can activate downstream AKT and ERK to drive cell proliferation and survival [34,35]. For FGF (fibroblast growth factor) signaling, when free FGF binds to FGFR, it activates the downstream PI3K/AKT pathway and exerts a protective effect on tight junction [36,37]. Additionally, for Wnt signaling, its binding to cell surface receptors can stabilize β-catenin levels and thereby modulating cell survival [38,39]. The cell metabolism module includes ATP-generating processes of aerobic and anaerobic glucose breakdown and several key enzymes involved in glucose metabolism such as TIGAR (TP53-induced glycolysis and apoptosis regulator) [40,41], PFKFB3 (6-phosphofructo-2-kinase), and the ATP sensor AMPK (5′-AMP-activated protein kinase) [[42], [43], [44], [45]]. The hypoxia regulation module explains the activation process of HIF1α (hypoxia-inducible factor 1 alpha) and HIF2α upon exposure to low oxygen, which further promotes the generation and secretion of cell viability-related proteins like Ang2, SEMA3G, and BDNF [26,27,[46], [47], [48], [49]]. Furthermore, activation of the HIF pathway regulates the downstream p53 (tumor protein p53)/BAX (BCL2-associated X)/Caspase3 cell apoptosis pathway [[50], [51], [52]]. The oxidative stress module focuses on the generation of ROS (reactive oxygen species) and ONOO^−^ and their effects on tight junction proteins [53,54] as well as their interactions with other signaling modules (e.g. the NFкB pathway) [19]. Moreover, we incorporated the release and exchange of calcium ions between cytosol and endoplasmic reticulum which are collectively regulated by VEGF signaling and ROS [55,56]. In summary, this integrative model informed by data-driven transcriptomic analysis of BMECs in stroke was able to mechanistically capture the complex structure and molecular interactions of a multipathway multifunctional cellular signaling system within BMECs.

### 2.2 Extensive model calibration and validation using pathway-specific and integrative cellular signaling data

We conducted a comprehensive process of model calibration and validation against experimental data. The BMEC model was first allowed to reach an unstimulated steady-state under normal oxygen and glucose conditions without external stimuli, and then an extensive set of experimental data measuring the cellular pathway response under different stimuli (e.g. ligands, stress) was simultaneously utilized for quantitative model calibration (a total of more than 300 data points). Subsequently, new experiments were performed and data were collected to validate the predictive capacity of the computational systems biology model.

**Fig. 2:**
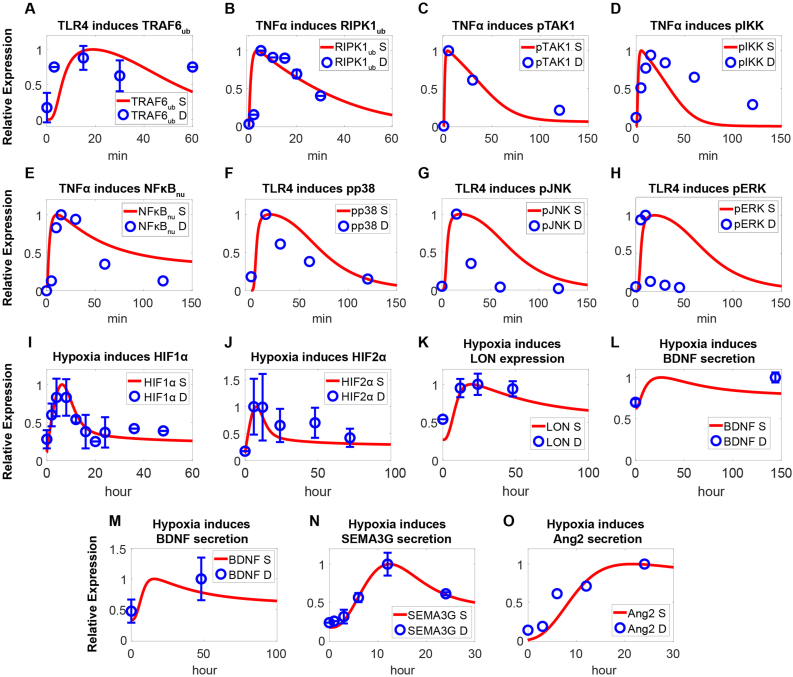
**Model calibration of inflammatory signaling and hypoxia regulation pathways in BMECs.** In the inflammation pathway module, **(A)** LPS (300 ng/ml) induces activation of TRAF6; **(B**–**E)** TNFα (1 μg/ml, 100 ng/ml, 200 ng/ml) induces ubiquitination and phosphorylation of **(B)** RIPK1, **(C)** TAK, **(D)** IKK, and **(E)** nuclear translocation of NFκB; **(F–H)** LPS (10 ng/ml, 100 μg/ml, 1 μg/ml) induces potent phosphorylation of downstream **(F)** p38, **(G)** JNK, and **(H)** ERK. In the hypoxia regulation module, **(I**–**J)** low oxygen (1 % O_2_) induces cellular accumulation of **(I)** HIF1α and **(J)** HIF2α. **(K–O)** Hypoxia (1 % O_2_, 0.5 % O_2_) upregulates **(K)** Lon expression, **(L**–**M)** BDNF secretion, **(N)** SEMA3G (mRNA) expression, and **(O)** Ang2 secretion. Y axes are relative expression levels (normalized to their respective maximum values). S, simulation; D, experimental data.

**Fig. 3:**
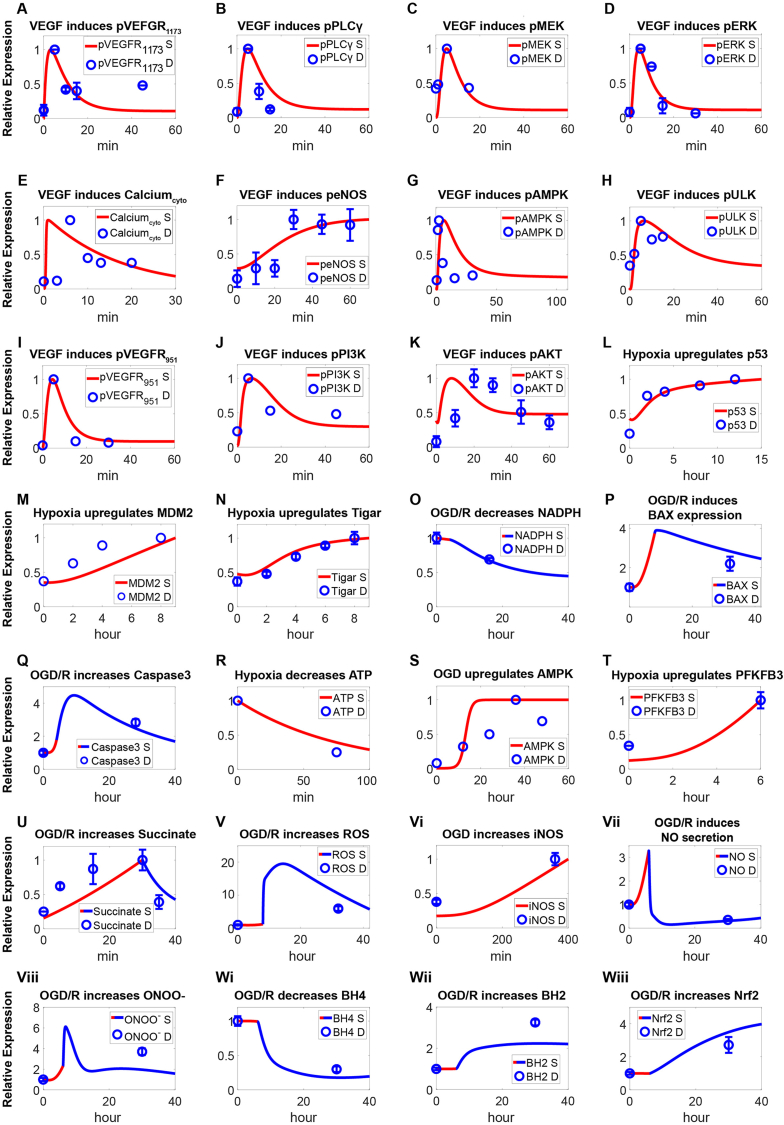
**Model calibration of cell survival and cell metabolism pathways in BMECs.** In growth factor-mediated cell survival pathways, **(A**–**D)** VEGF (50 ng/ml, 20 ng/ml) induces phosphorylation of **(A)** VEGFR at Y1173, and downstream phosphorylation of **(B)** PLCγ, **(C)** MEK, **(D)** ERK. **(E**–**H)** Activated PLCγ can lead to elevation of **(E)** cytoplasmic calcium and subsequently activate **(F)** eNOS, **(G)** AMPK, and **(H)** ULK. **(I–K)** VEGF (50 ng/ml, 20 ng/ml) also induces phosphorylation of **(I)** VEGFR at Y951, which leads to phosphorylation of **(J)** PI3K and **(K)** AKT. **(L**–**O)** In the cell survival module, hypoxia (3 % O_2_, 0 % O_2_, 1 % O_2_) can lead to the elevation of **(L)** p53, **(M)** MDM2, and **(N)** TIGAR, which further regulates **(O)** NADPH to counter oxidative stress. **(P**–**Q)** Under OGD/R conditions, expression of **(P)** BAX and **(Q)** Caspase3 were both upregulated, leading to cell apoptosis. **(R**–**T)** In the cell metabolism module, oxygen deprivation can lead to reduced **(R)** ATP production, thereby promoting the phosphorylation activation of **(S)** AMPK. Simultaneously, it upregulates the enzyme **(T)** PFKFB3 which is involved in ATP generation. **(U-Wiii)** Under OGD/R, cells differentially regulate **(U)** succinate in a time-dependent manner, which leads to elevation of **(V)** ROS. OGD also activates downstream **(Vi)** iNOS, resulting in generation of **(Vii)** NO and accelerating the production of **(Viii)** ONOO^−^. ROS also oxidizes **(Wi)** BH4 to **(Wii)** BH2, and can induce the expression levels of **(Wiii)** NRF2. Y axes are relative expression levels (normalized to their respective maximum levels or initial levels at control condition). S, simulation; D, experimental data. In OGD/R-treated group, red line represents OGD duration and the blue line represents R duration.

Our mechanism-based systems biology model was able to quantitatively capture the essential signaling processes involving numerous different BMEC functions (as detailed below), and the model results were consistent with literature reports. Model simulations of the inflammatory pathway revealed that following activation, both receptors propagated signals to downstream hubs, with activated TLR4 promoting the ubiquitination of TRAF6 (Fig. 2A, Fig. S1B) and activated TNFR (Fig. S1A) promoting the ubiquitination of RIPK1 (Fig. 2B, Fig. S2C) [[57], [58], [59]]. In their active states, both proteins facilitate the phosphorylation and activation of the downstream TAK1/IKK signaling pathway (Fig. 2C–D, Figs. S1D–S1G), leading to the dissociation and activation of IκB and NFκB (Fig. 2E, Figs. S1H–S1L) [[60], [61], [62], [63]]. NFκB also upregulates the expression of A20 (Fig. S1K) to negatively regulate the above pathway via feedback. Ultimately, liberated NFκB can translocate to the cell nucleus and induce cytokine secretion. Moreover, phosphorylated TAK1 also activates the p38/JNK/ERK (Fig. 2F–H, Fig. S1N) signaling pathway [64,65]. For the hypoxia regulation module, reduced enzymatic activity of PHD (prolyl hydroxylase domain) under hypoxia results in increased accumulation of HIF1α (Fig. 2I, Figs. S1Q–S1S) and HIF2α (Fig. 2J) [66,67]. HIFs can then translocate into the nucleus and transcriptionally upregulate Lon protease (Fig. 2K) which has a profound impact on ROS regulation [68]. Moreover, activated HIFs also enhance the endothelial secretion of pro-growth factors such as BDNF (Fig. 2L–M), SEMA3G (Fig. 2N) and Ang2 (Fig. 2O) to maintain tissue homeostasis [25,26,69,70]. In the cell survival module, binding of the pro-growth signal VEGF to the endothelial cell surface VEGFR leads to phosphorylation at Y1173 (Fig. 3A, Fig. S1T) and subsequent downstream phosphorylation of PLCγ/MEK/ERK (Fig. 3B–D, Fig. S1U) proteins [[71], [72], [73]]. Activated PLCγ facilitates the release of calcium ions from the endoplasmic reticulum into the cytoplasm (Fig. 3E, Fig. S1V), and these calcium ions interact with calmodulin to promote the phosphorylation and activation of eNOS (endothelial nitric oxide synthase) (Fig. 3F, Fig. S1W) [74,75]. Moreover, activated PLCγ can also induce phosphorylation of AMPK and ULK (UNC51 like kinase) (Fig. 3G–H) [76,77]. In addition, VEGF-induced phosphorylation of VEGFR at Y951 (Fig. 3I) activates downstream PI3K/AKT (Fig. 3J–K, Fig. S1X) pathway [71,78,79]. Wnt can stabilize β-catenin levels, as expected (Fig. S2S) [38,80]. Regarding Ang-Tie interaction, free Ang1 binding to Tie2 can increase Tie2 phosphorylation and subsequently enhance downstream phosphorylation of ERK and AKT (Figs. S2A–S2C) [34,35]. For regulation of cell survival, hypoxia-induced accumulation of HIFs activates downstream p53 (Fig. 3L, Figs. S2D–S2F) [81]. p53, in a negative feedback manner, promotes the generation of MDM2 proteins (Fig. 3M), which are known to inhibit p53 function [82]. Moreover, as shown by the model, p53 can stimulate the generation of TIGAR (Fig. 3N) to increase NADPH (Fig. 3O) levels and thereby mitigate ROS-related cellular oxidative stress [83,84]. Furthermore, p53 also activates modulators directly associated with cellular apoptosis, specifically the downstream proteins BAX (Fig. 3P, Fig. S2G) and Caspase3 (Fig. 3Q) [52,85].

**Fig. 4:**
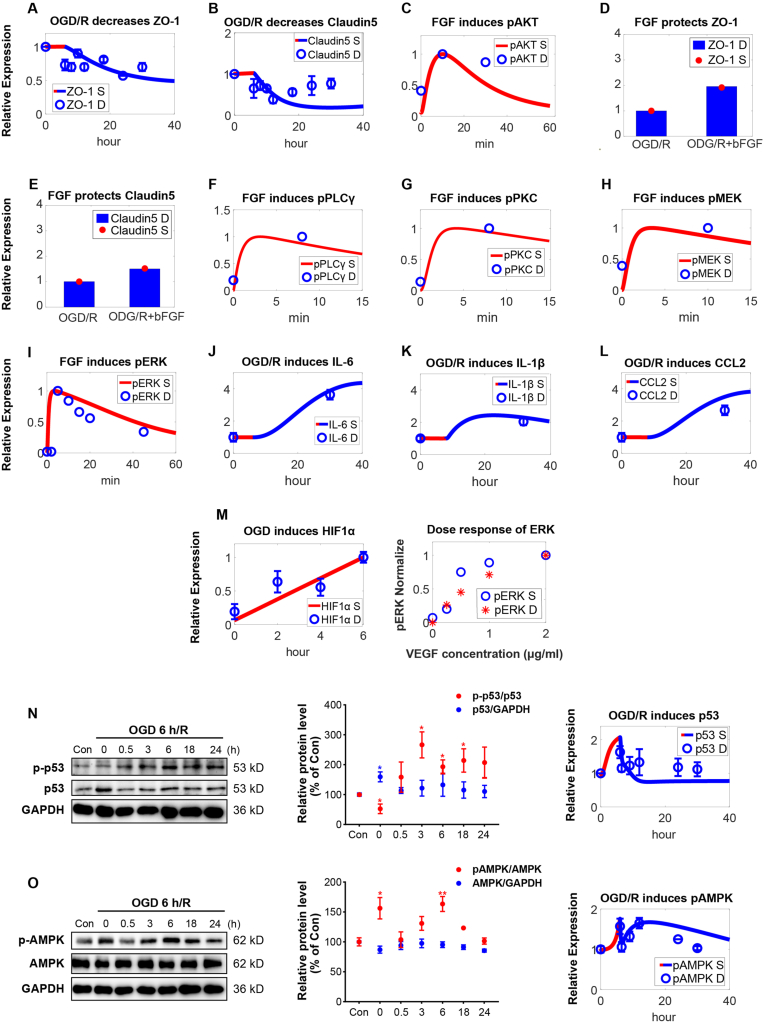
**Model-based integrative description of cellular stress response and experimental validation under OGD/R conditions. (A**–**B)** OGD/R results in significant downregulation of **(A)** ZO-1 and **(B)** Claudin5 in BMECs. FGF (1 μg/ml) can activate **(C)** AKT to protect tight junction damage regarding **(D)** ZO-1 and **(E)** Claudin5 under OGD/R. FGF (10 ng/ml, 1 μg/ml) also activates the **(F–I)** PLCγ/PKC/MEK/ERK axis to enhance cellular proliferation. **(J**–**L)** Under OGD/R conditions, ROS promotes the cellular secretion of inflammatory factors **(J)** IL-6, **(K)** IL-1β, and chemokine **(L)** CCL2. **(M)** OGD induces HIF1α level change in BMECs; the dose-response relationship of pERK under varying VEGF concentrations. **(N–O)** To validate the model, western blotting was used to analyze the cellular protein expression of two key modulators, p53 and pAMPK, in a BMEC cell line (bEnd.3) after OGD/R. Experimental data were then quantified and expressed as mean ± SEM (∗P < 0.05, ∗∗P < 0.01 vs Control, n = 3) and were directly compared with model simulations, suggesting overall good agreement from both quantitative and temporal perspectives. Y axes are relative expression levels (normalized to respective maximum levels or initial levels at control condition). S, simulation; D, experimental data. In OGD/R-treated group, red line represents OGD duration and the blue line represents R duration.

The cell metabolism module describes the shift in glucose metabolism when oxygen and glucose supplies are insufficient. Model simulations suggested that this shift would result in reduced generation of intracellular ATP (Fig. 3R and S2H) and increased AMPK activation (Fig. 3S, Fig. S2I) [86,87]. Furthermore, as shown by the model, the expression of PFKFB3, a key protein involved in glucose metabolism, is also upregulated under hypoxic conditions (Fig. 3T, Fig. S2J) [88]. In the oxidative stress module, model simulations showed that under OGD conditions, the accumulation of succinate within the cell first occurs due to reversed enzymatic activity of SDH (succinate dehydrogenase), and then upon reoxygenation, succinate was rapidly oxidized (Fig. 3U), leading to reverse electron transport and a rapid increase in ROS levels (Fig. 3V) [20,75,89]. Excessive ROS can enhance the activity of iNOS (inducible nitric oxide synthase) (Fig. 3Vi) and lead to the rapid generation of NO (Fig. 3Vii) in endothelial cells, which can further produce ONOO^−^ when combined with superoxide anions (Fig. 3Viii, Figure S2K) [5,90]. Cellular accumulation of ROS also oxidizes BH4 to BH2 (Fig. 3Wi, Fig. 3Wii) which can further intensify ROS accumulation [90]. Elevated ROS also activates the Keap1 (Kelch like ECH associated protein 1)/NRF2 (nuclear factor erythroid 2-related factor 2) feedback pathway (Fig. 3Wiii) [90], allowing NRF2 to mitigate excessive ROS through various mechanisms. Moreover, high levels of ROS can damage the tight junction proteins ZO-1 and Claudin5 (Fig. 4A and **4B**, Fig. S2L), leading to compromised tight junctions and increased BBB permeability [91,92]. Under these conditions, binding of extracellular FGF to FGFR can activate AKT (Fig. 4C) and protect tight junction (Fig. 4D and **4E**) during OGD/R [37,93,94]. Additionally, activated FGFR stimulates the PLCγ/PKC/MEK/ERK axis (Fig. 4E–I) to enhance cellular survival [[95], [96], [97]]. Increased ROS under OGD/R also mediates the upregulated secretion of inflammatory factors, such as IL-6 (Fig. 4J), IL-1β (Fig. 4K), and chemokine CCL2 (Fig. 4L, Fig. S2N), through NFκB (Fig. S2M) [90,98,99].

Regarding model validation, we first compared the model simulation with independent data that were not used during calibration, including the changes of HIF1α protein expression (Fig. 4M) in BMECs under OGD conditions, the dose-response curves for ERK, AKT and HIF1α (Fig. 4M, Figs. S2O–S2P) as well as data on NRF2-ROS feedback (Fig. S2Q), and the results were in good qualitative and quantitative agreement [100,101]. Then, we performed new *in vitro* experiments using bEnd.3 cells under OGD/R conditions and measured the time-course dynamics of two master regulators, p53 and AMPK. Under the same OGD/R conditions *in silico*, our model simulations of p53 expression (Fig. 4N) quantitatively matched the experimental data and demonstrated that p53 expression first increased under OGD conditions and then decreased upon reoxygenation. For AMPK, our model simulation of dynamic AMPK phosphorylation was also in good agreement with the experimental data and the unexpectedly observed two-peak phenomenon (Fig. 4O): under OGD, pAMPK levels increased significantly but would rapidly decrease to baseline upon reoxygenation, and later it would increase again and form a second peak around 6 h post-reoxygenation, then it would gradually decrease (e.g. dephosphorylated) until 24 h. These in-house experiments strengthened the predictive capacity of our computational model and confirmed the utility of the model in capturing the highly complex intracellular regulation in BMECs in response to combined OGD/R pathophysiological conditions.

### 2.3 Model-based dynamic characterization of functional marker expression in BMECs under various OGD/R conditions

**Fig. 5:**
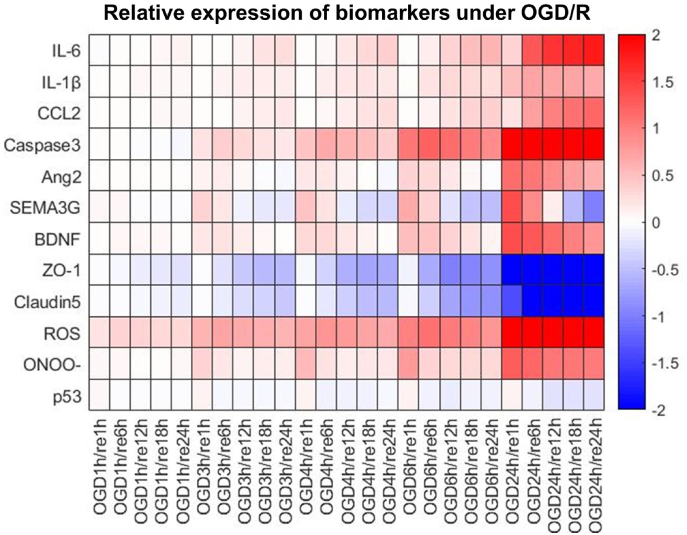
**Impact of different OGD/R conditions on key functional markers in BMECs.** Under various experimental OGD/R conditions, with different OGD times (1 h, 3 h, 4 h, 6 h, 24 h) and reoxygenation times (1 h, 6 h, 12 h, 18 h, 24 h, denoted as ‘re’), an expression change heatmap was generated to depict the relative dynamic changes in inflammatory cytokine secretion (IL-6, IL-1β, CCL2), growth factor secretion (Ang2, SEMA3G, BDNF), tight junction protein expression (ZO-1, Claudin5), generation of free radical species (ROS, ONOO^−^), and expression of apoptosis markers (p53, Caspase3) in BMECs. Relative expression fold changes of all markers were computed with respect to their initial expression under control condition (normoxia and normal glucose) and displayed in red-blue color map (values were log2 transformed).

To analyze the dynamic changes in the expression of key functional proteins and molecules in BMECs under different OGD/R conditions, we simulated 5 OGD durations (1 h, 3 h, 4 h, 6 h and 24 h) and 5 reoxygenation periods (1 h, 6 h, 12 h, 18 h and 24 h) and recorded the relative expression changes of 12 different functional markers, including inflammatory cytokines, growth factors, apoptosis markers, tight junction proteins, and free radicals (Fig. 5). The overall results revealed that the durations of OGD and reoxygenation can significantly influence cell viability and the functional phenotype of BMECs. Specifically, the secretion of inflammatory cytokines/chemokines such as IL-6, IL-1β, and CCL2 was positively correlated with the duration of OGD, and this trend did not diminish with increasing reoxygenation time. The damage to tight junction proteins caused by oxidative stress accumulated with prolonged periods of OGD and reoxygenation. Secretion of pro-growth factors such as Ang2, SEMA3G, and BDNF increased significantly with increasing OGD durations, but this effect tended to gradually return to baseline after reoxygenation as the underlying mechanism is largely driven by HIF activation. Additionally, we investigated the cellular generation of ROS and ONOO^−^ and found that with increasing OGD time, regardless of reoxygenation, the levels of these free radicals remained high (particularly for ROS). For cell apoptosis markers such as Caspase3, for short OGD durations (e.g. 3 h), its expression level would first increase then decline gradually with increasing reoxygenation time, but for long OGD durations (e.g. 24 h), Caspase3 protein expression remained at high levels without significant changes during reoxygenation, indicating that BMECs may enter an irreversible apoptotic state during long-term OGD. The simulation results demonstrated that the initial upregulation of p53 protein expression was minimally affected by OGD duration and it tended to decrease to stable levels close to or slightly below baseline after reoxygenation. These findings highlight that our model can be employed for the quantitative and dynamic analysis of the comprehensive condition- and time-dependent changes of BMEC functional phenotypes under various OGD and reoxygenation durations, providing new insights into the understanding of BMEC fate after stroke and reoxygenation/reperfusion. We also conducted simulations under other conditions incorporating different concentrations of TNFα and VEGF along with varying durations of OGD, as shown in Supplementary Figs. S3 and S4.

### 2.4 Identifying potential therapeutic targets through sensitivity analyses and *in silico* pharmacological intervention in model-based virtual BMECs

**Fig. 6:**
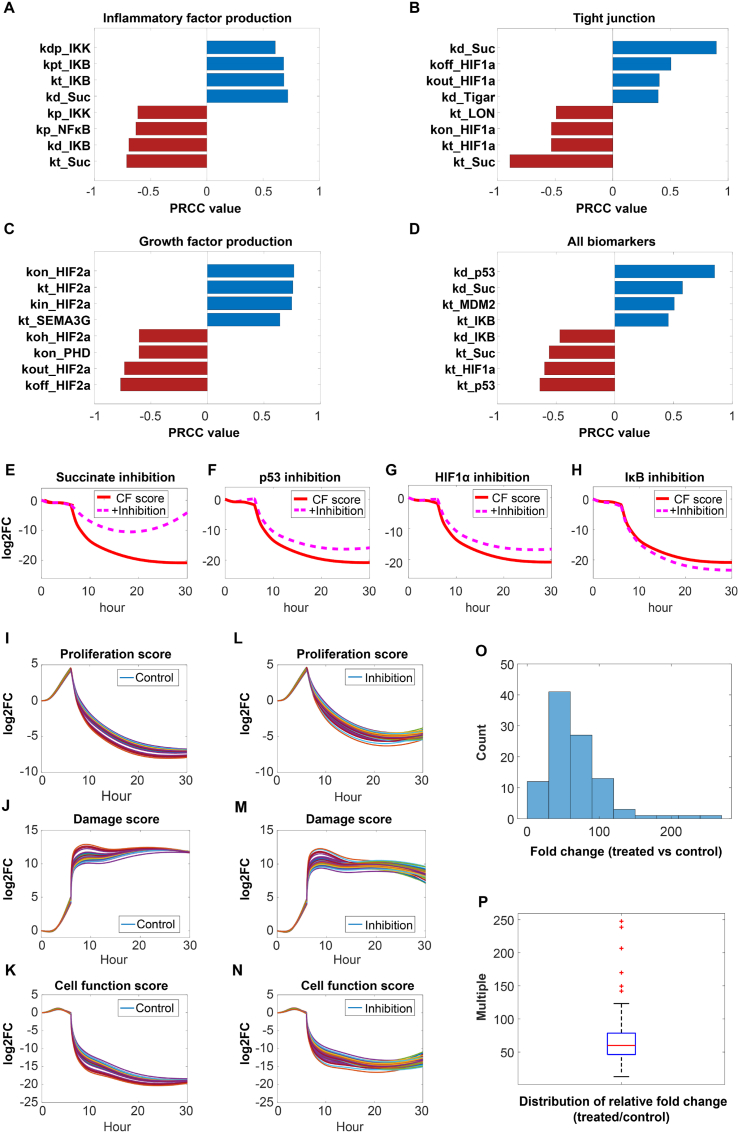
**Model-based analyses enable identification of potential therapeutic targets to reverse detrimental BMEC functional phenotypes under OGD/R.** Through sensitivity analysis, we identified the parameters that have the most significant impact on downstream biomarkers under OGD/R conditions (OGD-6 h, reoxygenation-24 h). The analyses were carried out in four parts, including sensitivities with respect to **(A)** inflammatory factor production, **(B)** tight junction, **(C)** growth factor production, and **(D)** total functional biomarkers. Subsequently, we conducted model simulations that intervened the top-ranked targets derived from the sensitivity analysis and evaluated their respective impact on downstream biomarker expression (toward more versus less protective directions), which include **(E)** succinate inhibition, **(F)** p53 inhibition, **(G)** HIF1α inhibition, and **(H)** IκB inhibition. We use cell function (CF score) as the final output. To further validate the findings across different BMECs, we simulated a cohort of 100 virtual cells under the same OGD/R conditions. These cells were assessed for **(I)** proliferation score, **(J)** damage score, and the **(K)** overall cell function score over time (see Methods for scoring details). **(L**–**N)** Additionally, inhibition of succinate was simulated in the virtual cells and the BMEC proliferation, damage, and cell function scores were computed and displayed. **(O–P)** Under OGD/R, the relative fold changes of the overall cell function scores of the BMEC cohort with succinate inhibition compared to scores of control BMECs were computed and displayed in histograms and boxplots, which indicated significant upregulation toward the direction of BMEC protection.

Following ischemic stroke, the most significant biological factor impacting BMEC function and fate is ischemia-reperfusion. We therefore selected the corresponding *in vitro* OGD/R conditions for the model sensitivity analyses to evaluate the regulatory potential of different intracellular targets on BMEC function. First, we specifically focused on the ability of BMECs to produce inflammatory and chemotactic factors as the primary output of interest in the sensitivity analysis, and the most sensitive parameters include those relevant to the NFκB pathway components (e.g. kp_IKK(ROS), kp_NFκB, kd_IKB(mrna), kt_Succinate) and ROS generation (e.g. kt_Succinate, kd_Succinate) (Fig. 6A). The expression of tight junction proteins can regulate BBB permeability; therefore, for this physiological aspect, parameters related to ROS generation (e.g. kt_Succinate, kd_Succinate) and HIF activity were suggested by the sensitivity analysis (e.g. kon_HIF1α, kt_HIF1α) as the most influential contributors (Fig. 6B). BMECs can also produce an array of pro-growth factors (e.g. SEMA3G, Ang2, BDNF) to counteract ischemic stress, and parameters that can significantly modulate HIF activation were suggested by the analysis (e.g. koh_HIF2α, kin_HIF2α) (Fig. 6C).Then, a comprehensive sensitivity analysis including the above three aspects of BMEC function together as the output of interest revealed that intracellular nodes such as succinate, IκB, p53, and HIF1α might be critical for the regulation of BMEC function and fate under OGD/R conditions (Fig. 6D). Therefore, we simulated a standard BMEC and the resulting cell function changes (using a computed cell function score considering 11 biomarkers, see Methods for details) if the above targets were pharmacologically modulated. Model simulations indicated that compared to untreated control under OGD/R, reducing the cellular availability of succinate (Fig. 6E), p53 (Fig. 6F), or HIF1α (Fig. 6G) can effectively increase and drive the overall cell function scores towards the pro-survival and less detrimental direction. Inhibition of IκB synthesis leads to increased NFκB signaling and enhanced secretion of inflammatory factors, thereby reducing the overall cell function score (Fig. 6H), and increasing IκB protein synthesis drives the cell response in the opposite direction (Fig. S2R). The complete sensitivity analyses results can be found in Supplementary Figs. S5–S8.

To further investigate the potential variation of BMEC functional phenotype response in a cell-by-cell manner, we generated 100 samples of the model, each representing a different single BMEC. These cells shared the same physiological mechanisms and pathway structures but had different parameter values determined by the sensitivity analysis to approximate the physiological heterogeneity of a real BMEC population *in vitro* [102]. To provide a comprehensive depiction of BMEC physiology at the population level, we conducted modeling analyses using three quantitative metrics (see Methods for details) in these virtual cells under OGD/R: the proliferation score (Fig. 6I), the damage score (Fig. 6J), and the aforementioned overall cell function score (Fig. 6K). From the simulations, we observed that the degrees of cell function changes under OGD/R were highly variable given the reasonable differences in the signal transduction rates in all individual cells. As our sensitivity analyses indicated the importance of succinate availability in modulating cellular response under OGD/R, we aimed to validate this finding in our virtual BMEC population. The cell population-level simulations demonstrated that the inhibition of succinate availability would give rise to superior cellular proliferation scores (Fig. 6L), lower damage scores (Fig. 6M), and overall better cell function scores (Fig. 6N) in the vast majority of cells than in the control OGD/R cells. Notably, the pooled virtual BMECs generally exhibited significant increase in overall cellular function scores (Fig. 6O and **6P**) after enforced inhibition of succinate availability, suggesting that targeting succinate may be a novel therapeutic route for alleviating microvascular damage in stroke. Furthermore, we simulated the performance of virtual cell populations in which HIF1α and p53 were suppressed respectively and analyzed their relative regulatory strengths (Figs. S9–S10). In conclusion, the above *in silico* analyses demonstrated the multifaceted potential of using our mechanistic computational model in the analysis of complex pathway dynamics and assessment of new drug targets in BMECs for ischemic stroke translational research.

### 2.5 *In vitro* experimental validation confirms that targeting succinate is an effective route to enhance BMEC protection under OGD/R conditions

**Fig. 7:**
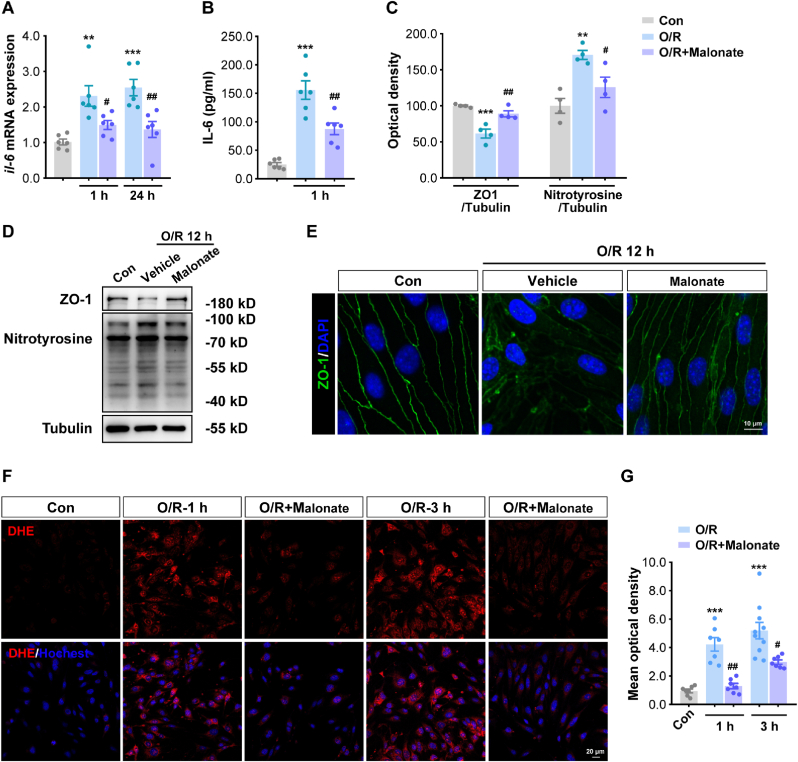
**Targeting succinate could protect BMECs from OGD/R insult *in vitro* as predicted by the systems biology model. (A)** Quantitative RT-PCR analysis of IL-6 mRNA in bEnd.3 cells after OGD/R and with malonate treatment. ∗∗P < 0.01, ∗∗∗P < 0.001 vs Con; #P < 0.05, ##P < 0.01 vs O/R (n = 6). **(B)** ELISA analysis of IL-6 secretion from bEnd.3 cells 1 h after reoxygenation. Data were expressed as mean ± SEM. ∗∗∗P < 0.001 vs Con; ##P < 0.01 vs O/R (n = 6). **(C)** Relative expression levels of ZO-1 and protein nitrotyrosine in bEnd.3 cells at 12 h after reoxygenation (in OGD/R) and with malonate treatment, and **(D)** corresponding Western blot results (tubulin was used as loading control). ∗∗P < 0.01, ∗∗∗P < 0.001 vs Con; #P < 0.05, ##P < 0.01 vs O/R (n = 4). **(E)** Immunofluorescence staining of ZO-1 (green) and DAPI (blue) in bEnd.3 cells at 12 h after reoxygenation and with malonate treatment (scale bar = 10 μm). **(F)** Representative photographs of DHE fluorescence staining of ROS (red) in bEnd.3 cells at 1 h and 3 h after reoxygenation and with malonate treatment, with quantified mean optical densities shown in **(G)** where data were quantified and expressed as mean ± SEM. ∗∗∗P < 0.001 vs Con; #P < 0.05, ##P < 0.01 vs O/R (n ≥ 7); scale bar = 20 μm. Statistical analyses were performed using one-way ANOVA. Abbreviations: O/R – OGD/R.

As our modeling analyses predicted a pivotal role of succinate availability and function in the decision of BMEC fate following ischemic insults, we then aimed to validate this finding experimentally by using a cell-permeable inhibitor, malonate, which can block succinate oxidation by SDH (and therefore can achieve a net effect very similar to limiting succinate availability and functioning as suggested by our computational model). We first found that the mRNA level of IL-6, a major pro-inflammatory cytokine expressed and secreted by BMECs, increased significantly in bEnd.3 cells after OGD/R, and that malonate treatment can significantly inhibit IL-6 mRNA expression (Fig. 7A). In addition, the exposure of BMECs to OGD/R insult resulted in a marked increase in cellular IL-6 secretion into the cell culture media, and this effect can also be suppressed by malonate (Fig. 7B). From the perspective of tight junction protection, malonate was shown to block the downregulation of ZO-1 protein expression in bEnd.3 cells induced by ischemic insult (Fig. 7C and **7D**); this finding was further supported by immunofluorescence staining as malonate treatment improved the expression and linear distribution of ZO-1 protein in the cell membrane (Fig. 7E). We then examined the effect of malonate on ROS and RNS, which are key endogenous drivers of cell damage in BMECs. We found that the expression of protein nitrotyrosine (reflecting RNS abundance) in bEnd.3 cells increased markedly upon OGD/R injury, and that malonate partially inhibited this increase (Fig. 7C and **7D**). For ROS, OGD/R significantly increased cellular ROS levels as determined by dihydroethidium (DHE) staining in bEnd.3 cells, and malonate treatment significantly repressed the cellular formation of ROS (Fig. 7F and **7G**). All these experimental results confirmed that targeting succinate, as predicted by our computational model, could effectively alleviate the damage to and reverse the detrimental phenotypes of BMECs under OGD/R from various physiological perspectives, which potentially provides a new therapeutic avenue for BBB protection in brain ischemia.

### 2.6 *In vivo* experiments in tMCAO mice further confirm that targeting succinate could reduce BMEC damage and brain injury

**Fig. 8:**
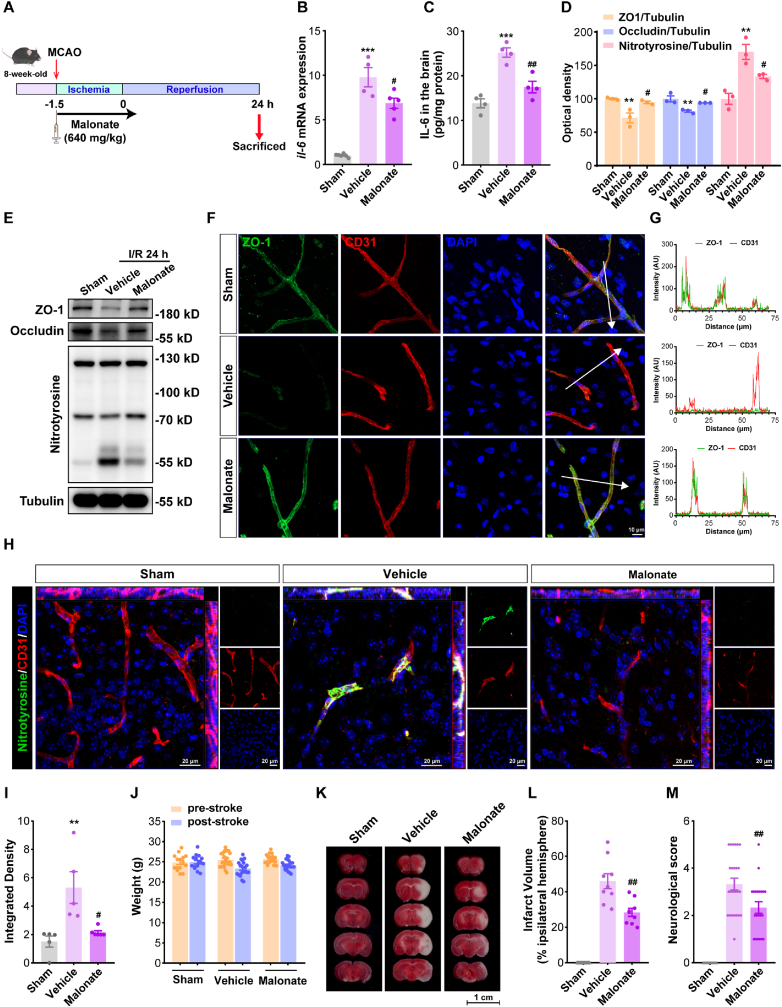
**Targeting succinate can help protect brain endothelium against ischemia/reperfusion injury and reduce brain damage in the mouse tMCAO stroke model. (A)***In vivo* experimental protocol of malonate treatment in mice exposed to tMCAO/reperfusion. **(B)** Quantitative RT-PCR analysis of IL-6 mRNA in cerebral microvascular endothelial cells isolated from mice at 24 h after I/R injury with or without malonate treatment. ∗∗∗P < 0.001 vs Sham; #P < 0.05 vs Vehicle (n = 4–6). **(C)** Measurement of IL-6 secretion in the cerebral tissue of tMCAO mice 24 h after reperfusion using ELISA. ∗∗∗P < 0.001 vs Sham; ##P < 0.01 vs Vehicle (n = 4). **(D)** Relative expression levels of ZO-1, Occludin and protein nitrotyrosine in cerebral tissue of tMCAO mice treated with or without malonate at 24 h after reperfusion, and **(E)** corresponding Western blot results (tubulin was used as loading control). ∗∗P < 0.01 vs Sham; #P < 0.05 vs Vehicle (n = 3). **(F)** Representative immunofluorescence staining of ZO-1 (green), CD31 (red) and DAPI (blue) in ischemic cerebral tissue of tMCAO mice treated with or without malonate at 24 h after reperfusion (scale bar = 10 μm). **(G)** Visual plots of the fluorescence intensity of ZO-1 and CD31 co-localization (in arbitrary units, AU) along the white arrows directions shown in **(F)**. **(H)** Representative immunofluorescence staining of nitrotyrosine (green) and CD31 (red) in cerebral tissue of tMCAO mice treated with or without malonate at 24 h after reperfusion, with quantified integrated densities shown in **(I)**. ∗∗P < 0.01 vs Sham; #P < 0.05 vs Vehicle (n = 5); scale bar = 20 μm. **(J)** Body weight of tMCAO mice with or without malonate treatment before ischemia and 24 h after reperfusion (n ≥ 10). **(K)** Representative TTC staining of coronal sections from tMCAO mice with or without malonate treatment at 24 h after reperfusion and **(L)** quantitative analyses of cerebral infarct sizes. Infarct volumes (%) were calculated as infarct area/total hemisphere for each mouse. ##P < 0.01 vs Vehicle (n = 9). **(M)** The neurological deficit scores of tMCAO mice were analyzed by Longa test at 24 h after reperfusion. ##P < 0.01 vs Vehicle (n ≥ 10). Data were expressed as mean ± SEM and statistical analyses were performed using unpaired *t*-test or one-way ANOVA. Abbreviations: I/R – Ischemia/Reperfusion.

To further validate our model prediction that targeting succinate is an effective route to enhance BMEC protection under ischemic conditions *in vivo*, we examined the impact of malonate on brain ischemia/reperfusion (I/R) injury in the mouse tMCAO stroke model. Mice were first exposed to tMCAO procedure for 90 min and malonate was administered via continuous intravenous infusion at a dose of 640 mg/kg. At 24 h after reperfusion, mice were sacrificed for further experimentation (Fig. 8A). Consistent with the aforementioned *in vitro* results, IL-6 mRNA levels in the dissected brain microvessels and overall IL-6 secretion were increased significantly in stroke mice at 24 h after reperfusion, and this trend was inhibited by malonate (Fig. 8B–C). In addition, consistent with our *in vitro* findings, malonate treatment was also found to prevent I/R-induced degradation of tight junction proteins *in vivo*, as seen by preservation of ZO-1 and Occludin compared to the vehicle group (Fig. 8D–E). Furthermore, double-labeling immunofluorescence also showed that the endothelial co-localization of ZO-1 and CD31 (microvessel marker) *in vivo* was markedly increased after malonate treatment compared to vehicle group (Fig. 8F–G). Next, dual-immunostaining for microvessels markers and nitrotyrosine revealed a significant increase in nitrotyrosine intensity, which indicates elevated nitrosative stress in mouse brain microvessels in response to I/R injury *in vivo*, and this trend was mitigated by malonate (Fig. 8H–I); similar results were also reported by western blotting data (Fig. 8D–E). Finally for post-stroke brain damage, malonate treatment can significantly alleviate cerebral infarction and neurobehavioral deficits in experimental stroke mice without affecting their weight (Fig. 8J–M). These *in vivo* experimental data from tMCAO mice again confirmed our systems model-based prediction regarding succinate as a potential endothelium-based drug target for ischemic stroke and demonstrated that this new route can effectively reverse the adverse BMEC phenotypes during I/R injury to achieve enhanced endothelial protection as well as reduced brain damage.

## 3 Materials and methods

### 3.1 Bioinformatics analysis of BMEC transcriptome

To investigate the signaling changes in BMECs after experimental ischemic stroke, cell transcriptome profiles were extracted from GEO dataset (GSE163752). Data measured from BMECs in tMCAO mice were selected specifically for further analysis. Differentially expressed genes (DEGs) (|log (2) Fold Change| > 1 and P value < 0.05) were identified from BMECs from ipsilateral (ischemic) versus contralateral (control) hemispheres in mice post tMCAO (transient middle cerebral artery occlusion) using the “DESeq2” R package. Gene set enrichment analysis (GSEA) and Gene ontology (GO) enrichment analysis were performed by R package “clusterProfiler”. Significant terms were identified with P.adjust <0.05. R package “emapplot” was used to conduct enrichment map from significant GO terms.

### 3.2 Summary of model formulation and parameter calibration

The computational systems biology model was developed based on ordinary differential equations (ODEs) and consists of a total of 108 molecular species and 145 reactions. Details regarding model initial conditions, equations and parameters can be found in the supplementary materials (Table S1 and Fig. S11). The entire model structure and pathway modules were designed and selected based on outputs from the transcriptomic analysis and extensive literature survey. All model data, including reaction rules, species, reaction rates, and parameter values, were processed using MATLAB's Simbiology Toolbox (MathWorks, Natick, MA). Model simulations were performed using the ode15s solver. As our model simulates a variety of ligand stimulation conditions, we converted all dosing units used in *in vitro* experiments (often in ng/ml) into standardized number of molecules per cell. For a particular ligand, experimental protocol information was extracted (e.g. cell culture density, dose), and the converted ligand doses were computed using the corresponding protocol information and ligand molecular weights (divide the total number of ligands by the average number of cells per unit volume). In the hypoxia regulation module and cell metabolism module, assumptions were made regarding the initial amounts of oxygen and glucose, as well as their changes following hypoxia and glucose deprivation. Based on experimental knowledge, it was determined that the standard oxygen concentration in the cell culture incubator was 21 % O_2_, reflecting indoor air conditions, and oxygen in OGD was close to 0 %. Glucose concentration for normal BMEC culture conditions was determined to be 5.05 mmol/L in our model, which is consistent with commonly used experimental protocols. To simulate the state of hypoxia and glucose deprivation followed by their restoration, the initial levels of oxygen and glucose within the cells were set to close zero. This simulated condition was maintained for a period of time (e.g. OGD duration), and all resulting cellular signaling changes were computed and recorded. Subsequently, oxygen and glucose levels were restored as in experiments, and the previously recorded cell species states at the end of OGD were applied as the new initial conditions to simulate the resulting changes in cell signaling during the reoxygenation (R) period. ImageJ software (NIH) was utilized in the analysis and quantitative digitization of Western blot and other experimental datasets. Initial concentration values of different proteins (mostly in copy numbers) and model species half-lives, turnover rates, phosphorylation rates, and certain receptor-ligand association and dissociation rates were calculated based on data reported by literature [[103], [104], [105], [106]]. In terms of building a BMEC *in vitro* steady state, we computationally enforced that all model species should not vary significantly during a substantial timespan under standard cell culture condition (e.g. control/untreated). For this, we identified a set of parameters and initial conditions to help ensure that all model species' concentrations would always reside within 0.9x and 1.1x fold of their respective reasonable literature-derived values during ∼7 days of control cell culture condition (no external perturbation) to mimic an equilibrium quiescent cell state. Then different stimuli such as inflammatory cues or OGD were applied on top of this quiescent state, and model simulations can be compared with corresponding experimental datasets. After a first round of manual module-by-module model calibration, automated global optimization using the patternsearch function in MATLAB (10000 iterations) was performed on all free parameters within a range of 0.5x to 2x fold of their derived values against all calibration datasets simultaneously (including different stimuli, different species, different timepoints, etc, with more than 300 datapoints in total).

### 3.3 Model sensitivity analysis

Model sensitivity analysis was conducted using the Latin Hypercube Sampling method to compute the Partial Rank Correlation Coefficient (PRCC) values within a parameter variation range of 0.5x to 2x using the algorithm provided by Marino et al. [107]. Specifically, we focused on the OGD/R conditions as the most relevant condition for experimental ischemic stroke, with OGD lasting for 6 h and reoxygenation for 24 h. The parameter variations were explored by running the simulation 5000 times. We assessed the impact of OGD/R and corresponding parameter sensitivities by analyzing the outputs of functional modules: inflammatory pathway module output was represented by[IL-6]∗[IL-1β]∗[CCL2]; growth factor module output was represented by[Ang2]∗[SEMA3G]∗[BDNF]; tight junction module output was represented by[ZO-1]∗[Claudin5]; and the combined cell function score under OGD/R was computed by dividing the "positive" cell proliferation score ([Ang2]∗[SEMA3g]∗[BDNF]∗[ZO-1]∗[Claudin5]) by the "negative" damage score ([IL-6]∗[IL-1β]∗[CCL2]∗[ROS]∗[ONOO^−^]∗[Caspase3]) and we normalize the values obtained for each species by dividing them by their initial values under control condition. Subsequently, we plotted the time-dependent profiles of each score as the final output.

### 3.4 Generation of model-based virtual single cells

The overall flow of creating model-based virtual cells is similar to that proposed in Zhao et al. [108]. To analyze the behavior of BMECs with physiological variability, we generated 100 virtual cells for analysis under OGD/R conditions. The virtual cells were subjected to the experimental conditions of OGD for 6 h followed by reoxygenation for 24 h. Based on the results from the PRCC analysis, we selected key parameters that can significantly influence cellular states and randomly assigned them values between 0.5x and 2x of their baseline using a normal distribution. To study the cellular states of virtual cells after succinate targeting, we further assigned the parameters associated with succinate synthesis and turnover with a value range of 0.5x to 0.8x of their baseline values to simulate variability in intervention efficacy. Similar to the sensitivity analysis, the outputs were then represented using the same cell function response metrics for each model-based individual cell (curve) and analyzed using histograms and boxplots to visualize distribution.

### 3.5 Cell culture

Mouse brain microvascular endothelial cells (bEnd.3) were grown in Dulbecco's modified Eagle's medium (DMEM, Gibco, USA) containing 10 % fetal bovine serum (FBS, Gibco, USA) and 1 % penicillin/streptomycin and maintained at 37 °C under an atmosphere containing 95 % O_2_ and 5 % CO_2_. The cells were passaged with trypsin (0.25 %)-EDTA (0.02 %) in PBS at a split ratio of 1:4 after reaching 80–90 % confluence.

### 3.6 *In vitro* oxygen-glucose deprivation and reoxygenation (OGD/R) and drug treatment

OGD/R model was used to mimic cerebral ischemia/reperfusion *in vitro* as described previously [109,110]. Briefly, bEnd.3 cells were seeded into a 6-well plate and cultured with DMEM medium in an incubator with 95 % air and 5 % CO_2_ at 37 °C before OGD/R. For the OGD procedure, the culture was washed twice with glucose-free Hank's balanced salt solution (HBSS, 116 mM NaCl, 5.4 mM KCl, 0.8 mM MgSO_4_, 1.0 mM NaH_2_PO_4_, 1.8 mM CaCl_2_, and 26 mM NaHCO_3_, pH 7.3), and then the cells were placed in an experimental hypoxia chamber (Stemcell, Canada) for 6 h, which was filled with 95 % N_2_ and 5 % CO_2_ (v/v). After OGD, the cultures were replaced with fresh culture medium. For dimethyl malonate treated group, the cell cultures were treated with 5 mM dimethyl malonate (MCE, China) for OGD and reoxygenation.

### 3.7 Transient middle cerebral artery occlusion (tMCAO) and reperfusion model of stroke in mice

Male C57BL/6 mice, 8-week-old, were purchased from GemPharmatech Co., Ltd (Nanjing, China) and housed under conditions of constant temperature and humidity, kept on a 12:12-h light:dark cycle (lights on 9–21 h), and fed ad libitum. All animals were used in accordance with the institutional guidelines for animal use and care and the study protocol was approved by the ethical committee of Nanjing Medical University (IACUC-2403063). MCAO surgery was performed as described previously [111,112]. Briefly, mice were anesthetized with isoflurane isoflurane (4 % induction, 1.5–2% maintenance) in O_2_. Next, a 6-0 suture with a silicone-coated tip (602223PK10RE, Doccol Corporation, USA) was inserted from the right external carotid artery to the internal carotid artery, extending to the origin of the middle cerebral artery. After an ischemia duration of 90 min, the suture was withdrawn to allow reperfusion. The mice in sham operation group were conducted with the same procedure, except for suture insertion. The body temperature of the mice was maintained at 37.0 ± 0.5 °C by a heating blanket during I/R operation. For dimethyl malonate treatment, the mice were infused with dimethyl malonate (640 mg/kg in total) by intravenous infusion 90 min during tMCAO and 10 min after reperfusion. Mice were sacrificed 24 h after reperfusion.

### 3.8 Western blotting

Western blotting analysis was carried out according to protocols as described previously [113]. Total proteins were extracted from bEnd.3 cells or ischemic penumbra of mouse brain tissue using RIPA lysis buffer containing phenylmethylsulfonyl fluoride (PMSF) and phosphatase inhibitor, The equivalent amount of protein was separated by 10 % acrylamide denaturing gels (SDS-PAGE), and then transferred to PVDF membrane (Millipore, Billerica, MA, USA). Membranes were blocked with fat-free milk for 1 h and incubated with primary antibodies as following: anti-*p*-AMPK (1:1000, #2531, CST, USA); anti-AMPK (1:1000, #2532, CST, USA); anti-p-p53 (1:2000, #2528, CST, USA); anti-p53 (1:1000, #2524, CST, USA); anti-ZO-1 (1:1000, 61–7300, Invitrogen, USA); anti-Nitrotyrosine (1:500, #05–233, Millipore, USA); anti-Occludin (1:1000, 71–1500, Invitrogen, USA); anti-GAPDH (1:5000, #2118, CST, USA) and anti-β-Tubulin (1:5000, T5201, Sigma-Aldrich, USA) at 4 °C overnight, and then incubated with HRP-conjugated secondary antibodies (1:5000, Life Science) at room temperature for 2 h. The images were captured with the Tanon™ 5200CE Chemi-Image System (Tanon, China). The density of the bands was quantified with ImageJ software (NIH, Bethesda, MA, USA) and normalized to corresponding loading control.

### 3.9 Isolation of mouse brain microvessels

Brain microvessels were isolated from individual mouse ischemic brains as described previously with modifications [114]. In brief, the cerebral ischemic brain was dissected and digested for 30 min at 37 °C on a rotator with 1 ml homogenization buffer (10 mM HEPES, 5 mM Ca^2+^, 400 U/mL collagenase Ⅱ (Solarbio), and 10 mg/mL DNase I (Solarbio) in DMEM) with gentle trituration every 10 min. Then, the homogenizer was centrifugated at 1000 g for 5 min at 4 °C. The cell pellets were resuspended in 20 % BSA and centrifuged at 1000 g at 4 °C for 20 min. Finally, the cell pellets were collected for further RNA extraction.

### 3.10 Real-time quantitative Polymerase chain reaction (RT-PCR)

Total RNA were extracted from bEnd.3 cells or mouse brain microvessels in the ischemic penumbra with RNAiso Plus (9109, TaKaRa, Japan), and used in reverse transcription as described previously [111]. Real-time PCR amplification was performed using AceQ qPCR SYBR Green Master Mix (Q131-02, Vazyme, China) following the manufacturer's protocol. The mRNA expression level was determined using the comparative threshold (Ct) method by normalizing the target mRNA Ct values to those for Actin (ΔCt). Statistical analysis of real-time PCR data was performed using 2^−ΔΔCt^ values [115]. The primer sequences used in this study were as follows:

Mouse *il-6* forward: 5′- ACAACCACGGCCTTCCCTAC-3′

Mouse *il-6* reverse: 5′- TCTCATTTCCACGATTTCCCAG-3′

Mouse *Actin* forward: 5′- AGATCAAGATCATTGCTCCTCCTG-3′

Mouse *Actin* reverse: 5′- GGGTGTAAAACGCAGCTCAG-3′

### 3.11 Measurement of secreted IL-6 levels

IL-6 concentrations were measured with the mouse IL-6 precoated ELISA kit (1210602, Dakewe, China) following manufacturer's instructions. *In vitro*, bEnd.3 cells were exposed to OGD/reoxygenation with or without 5 mM dimethyl malonate treatment. The medium from the bEnd.3 cells culture was collected 1 h after reoxygenation after centrifugation. *In vivo*, the lysates from ischemic penumbra tissues of mice were collected at 24 h after reperfusion. A450 was measured using a microplate reader (Bio Tek ELx800).

### 3.12 Immunofluorescence staining

*In vitro*, bEnd.3 cells were grown on coverslips in 24-well plates and treated with OGD for 6 h. *In vivo*, mice were anesthetized at 24 h after reperfusion and transcardially perfused with PBS (pH 7.4) followed by 4 % PFA in PBS immediately as described previously. Sections of the brains (45 μm thick) were cut with a freezing microtome (Leica CM1950). Then, the cells or brain sections were washed with PBS and fixed with 4 % PFA for 15 min followed by permeabilization for 5 min. After blocking with 5 % BSA for 1 h, the samples were incubated with primary antibody as following: anti-ZO-1 (1:300), anti-CD31 (1:300, AF3628, R&D, USA), anti-nitrotyrosine (1:500) at 4 °C overnight. After washing, the samples were incubated with secondary antibody for 2 h at room temperature. The nuclei were stained with DAPI (Vector Laboratories, Burlingame, CA) for 10 min. Immunofluorescence confocal microscopy was performed with a confocal laser scanning microscope (Olympus LSM800).

### 3.13 Measurement of infarct volume

At 24 h after reperfusion, the mice were sacrificed by cervical dislocation. Brains were dissected out and immediately cooled to −20 °C for 10 min. Then, brains were dissected on ice and sliced into five coronal sections at 2 mm thickness. Brain sections were incubated in 1 % 2,3,5-triphenyltetrazolium chloride (TTC; Sigma, USA) for 20 min at 42 °C, followed by overnight immersion in 4 % PFA. The infarct volume was calculated as sum of (infarct area × slice thickness) and presented as percentage of the healthy hemisphere volume by ImageJ software (NIH, Bethesda, Maryland, USA). When no infarct was detected, the percentage was indicated as 0.

### 3.14 Assessment of neurological deficits in mice

At 24 h after reperfusion, assessment of the neurological deficits in mice was carried out by an investigator blinded to the treatment groups to evaluate neuronal function impairment using the modified scoring systems (0–5) proposed by Longa et al. [116]. The following scale rating was used: 0, normal motor function; 1, forelimb flexion when lifted by the tail; 2, circling to the contralateral side when held by the tail on a flat surface but normal posture at rest; 3, leaning to the contralateral side at rest; 4, no spontaneous motor activity; 5, dead. The mice in the sham group exhibited no manifestations of neurological deficits.

### 3.15 Measurement of ROS in bEnd.3 cells

*In vitro* bEnd.3 cells were exposed to OGD for 6 h. Intracellular ROS were measured 1 h and 3 h after reoxygenation with incubation with 2 μM dihydroethidium (DHE, Sigma-Aldrich, USA) for 30 min at 37 °C in the dark [109]. The cells were then washed twice with PBS followed by incubation with hochest (62249, Thermo Fisher, USA). After washing with PBS twice, immunofluorescence confocal microscopy was performed with a confocal laser scanning microscope (Olympus LSM800).

### 3.16 Statistical analyses

All data are presented as mean values ± SEM. Either an unpaired *t*-test or one-way ANOVA (followed by Tukey's post hoc multiple comparison test) was used to compare the values between the groups. P values < 0.05 were considered statistically significant.

## 4 Discussion

We have here constructed and presented a large-scale computational systems biology model of the BMEC intracellular signaling network to describe its complex molecular response and functional changes in experimental stroke conditions. The essential pathway composition and structure of our model are mechanistically derived from comprehensive bioinformatics analyses of *in vivo* stroke-induced BMEC-specific transcriptome. Another advancement compared to previous modeling efforts is that our mechanistic BMEC model has been significantly enriched in terms of stroke-related pathway modules and regulatory axes and the model has been extensively calibrated and validated against multi-modal quantitative experimental datasets. By incorporating different physiological aspects of BMEC function in health and disease, our model can integratively predict the temporal changes of an array of BMEC functional markers during composite experimental stroke conditions such as OGD/R, which further allowed us to examine and validate new therapeutic targets that could potentially reverse the detrimental BMEC phenotype response that commonly occurs after stroke and reperfusion damage, with the ultimate goal of mitigating post-stroke brain damage [30]. In addition, as a crucial component in the neurovascular unit (NVU) which serves pivotal foundational roles in stroke pathophysiology, BMECs are capable of communicating with other NVU cell members such as neurons, astrocytes, microglia, pericytes as well as other peripheral immune cells [36,117,118]. A number of key growth factors, cytokines and chemokines used in such communication were already included in this model version; still, future efforts that model BMECs can further account for the various endothelial cell adhesion molecules (e.g. selectins) critical for the attraction and infiltration of neutrophils and T cells during stroke progression [119]. Recent studies that utilized molecular profiling of cerebral microvascular transcriptomics in experimental stroke conditions, such as the one by Callegari et al., can also serve as important resources for the identification of new neurovascular protection-related therapeutic pathways and targets [120], which as we envision will nicely complement future mechanistic modeling efforts in a synergistic manner.

Our model sensitivity analyses suggested that regulation of key targets including succinate, p53, and HIF may have significant impact on the functional response of BMECs under OGD/R conditions, particularly driving them toward the pro-survival and protective phenotypes. Through further model-based virtual cell analysis and screening, we first mechanistically predicted the therapeutic potential of targeting succinate in effectively reversing OGD/R-induced BMEC response. We then validated this prediction through *in vitro* OGD/R and *in vivo* tMCAO mouse experiments, finding that pharmacological inhibition of succinate (using malonate) can not only reduce cell secretion of inflammatory cytokines but also alleviate free radical generation and tight junction damage. Previous studies have shown that OGD can cause reversal of mitochondrial SDH activity and lead to substantial succinate accumulation within cells, and given the restored SDH activity after reoxygenation, extensive oxidation of accumulated cellular succinate can result in high production of ROS and initiate oxidative cell damage [20]. Malonate is a competitive inhibitor of succinate dehydrogenase. It binds to the active site of succinate dehydrogenase and inhibits the enzyme activity and thereby reducing the conversion of succinate to succinyl-CoA. In line with our model prediction and experimental results, other studies have also confirmed the beneficial role of malonate in various types of ischemia-reperfusion tissue injury [[121], [122], [123]]. However, malonate or any other SDH inhibitors have not yet entered the clinical trial stage, and research is still under way to optimize their *in vivo* delivery and function [124,125]. Another target identified from our integrative analysis is p53, a critical regulator of apoptosis and cell cycle [126] with substantial drug development efforts targeting p53 in the field of oncology [[127], [128], [129]]. Model simulations suggested that inhibiting p53 activity resulted in reduced expression of apoptotic markers in BMECs after OGD/R, which can be largely attributed to less downstream BAX/Caspase3 pathway activity. Consistent with this finding, many experimental studies have already confirmed the protective role of inhibiting p53 in ischemic stroke animals [130,131]. For HIFs which are key proteins in cellular hypoxia regulation and hot targets for drug development, previous efforts mainly focused on inhibiting HIFs to downregulate cell proliferation and compensatory growth factor secretion to combat cancer. Interestingly in our model, inhibiting HIF1α was predicted to drive the overall BMEC function response toward the pro-survival and protective directions in OGD/R, although in literature mixed results of how HIF1α perturbation would impact ischemic stroke tissue damage and recovery have been reported [[132], [133], [134]]. One possible explanation is that the temporal regulation of HIF1α in stroke is complex and it has many cell type-specific downstream targets in the brain.

BMECs were recognized as playing crucial roles not only in ischemic stroke pathophysiology but also in many other deleterious CNS diseases such as Alzheimer's disease [135], Parkinson's disease [136], and multiple sclerosis [137]. Often secondary to the onset of these brain diseases, BMEC signaling and function become abnormally altered as they continuously crosstalk with other brain cells and environmental cues. Such BMEC dysfunction is most commonly manifested as damaged tight junctions and increased blood-brain barrier permeability, which would physically allow passage of harmful substances into the CNS and further exacerbate the tissue microenvironment during disease progression [9,10]. Studies have shown that microglia can secrete inflammatory factors such as TNFα and IL-6 and cause damage to the blood-brain barrier in stroke [138], while certain CNS fibroblasts are involved in the repairing process of damaged tight junctions and management of infarct volume [139]. It has also been reported that co-culture of neurons with BMECs together could directly result in significant worsening of BBB function compared to BMEC culture alone under OGD/R, indicating potential stress-induced crosstalk between neurons and BMECs that could regulate BBB integrity [140]. Therefore, an immediate next-step to enrich our model utility is to incorporate the physiology of these different brain cells to collectively portray the multi-cellular tissue landscape and dynamic cell-cell interactions in ischemic stroke. This would enable a more comprehensive characterization of ischemic stroke disease progression for researchers to prospectively evaluate new pharmacological therapies in a high-throughput manner. Such mechanism-based multiscale modeling strategies have provided significant insights for the translational research of many major human diseases [[141], [142], [143]], but related efforts in ischemic stroke research is still nascent. In addition, we envision that our physiology-based models can also be combined with biophysics-type models (e.g. computational fluid dynamics) to create ischemic stroke patient digital twins and drive model-based translational applications in advanced clinical-level scenarios such as simulation of surgical trials and personalized medicine [144,145].

### 4.1 Limitations of the study

Our present study integrates transcriptomics-based bioinformatics analyses together with mechanism-based systems biology modeling to investigate key intracellular regulatory hubs that could effectively protect BMECs in adverse stroke-related experimental conditions. While the therapeutic potential of the model-predicted target, succinate, was successfully validated *in vitro* and *in vivo*, it should be noted that malonate, the commonly-used pharmacological agent to inhibit succinate accumulation and oxidation, might have additional off-target effects. It was recently reported that low-versus high-dose malonate can differentially regulate cell survival via p53-dependent pathways [146], and that the endogenous metabolite of malonate, namely malonyl-CoA, was also a potent factor that can influence mitochondrial metabolism [147]. Thus, further studies are needed to better understand the full picture of malonate in tuning succinate function and impacting cell survival. Regarding the overall analytical and modeling methods employed, we still envision that the current model scope and mechanistic details can be continuously refined and expanded in future when more stroke-related multi-omics data becomes available (especially brain microvasculature-specific data), including those from animal models and human patients [148,149]. In this sense, our BMEC model platform can serve as a starting basis from which future enrichment of the details and interactions within different pathway modules will enable us to more thoroughly examine the dynamic multiplex regulation and BMEC fate stimulated by stroke triggers and the pathophysiological microenvironment. As for our model-based BMEC phenotype readouts, while we currently used a mechanistic biomarker-based metric system (based on the relative expression profiles of numerous pro- and anti-survival/growth/inflammation molecules produced by BMECs), there is still a connection to be established between quantitative biomarker-based scoring and qualitative BMEC phenotypes, which would call for more in-depth studies through combined computational plus experimental approaches.

## CRediT authorship contribution statement

**Geli Li:** Writing – original draft, Software, Methodology, Investigation, Formal analysis. **Yuchen Ma:** Methodology, Investigation, Formal analysis. **Sujie Zhang:** Investigation, Formal analysis, Data curation. **Wen Lin:** Investigation, Formal analysis. **Xinyi Yao:** Investigation, Formal analysis, Data curation. **Yating Zhou:** Writing – review & editing, Investigation, Data curation. **Yanyong Zhao:** Writing – review & editing, Data curation. **Qi Rao:** Writing – review & editing, Formal analysis. **Yuchen Qu:** Writing – review & editing, Formal analysis. **Yuan Gao:** Writing – review & editing, Methodology, Investigation, Formal analysis. **Lianmin Chen:** Writing – review & editing, Methodology. **Yu Zhang:** Writing – review & editing, Methodology. **Feng Han:** Writing – review & editing, Conceptualization. **Meiling Sun:** Writing – review & editing, Writing – original draft, Conceptualization. **Chen Zhao:** Writing – review & editing, Writing – original draft, Validation, Supervision, Software, Resources, Methodology, Investigation, Funding acquisition, Conceptualization.

## Declaration of competing interest

The authors have declared that no competing interests exist.

## Data Availability

All data used for this research and modeling analyses were included in the manuscript and supplementary materials.
