# Supplementary material for: A mechanistic systems biology model of brain microvascular endothelial cell signaling reveals dynamic pathway-based therapeutic targets for brain ischemia
Source: Redox Biol. 2024 Nov 5;78:103415. doi: 10.1016/j.redox.2024.103415 (PMC11584692; doi:10.1016/j.redox.2024.103415)
Supplement: Multimedia component 1 [file mmc1.pdf]

Supplementary Materials

A mechanistic systems biology model of brain microvascular endothelial cell signaling reveals dynamic pathway-based therapeutic targets for brain ischemia

Geli Li[1,2], Yuchen Ma[1], Sujie Zhang[1], Wen Lin[1], Xinyi Yao[1], Yating Zhou[3], Yanyong Zhao[1], Qi Rao[1], Yuchen Qu[1], Yuan Gao[4], Lianmin Chen[3], Yu Zhang[5], Feng Han[6*], Meiling Sun[7*], Chen Zhao[1,3*]

1 School of Pharmacy, Nanjing Medical University, Nanjing, China, 210000

2 Gusu School, Nanjing Medical University, Suzhou, China, 215000

3 The First Affiliated Hospital of Nanjing Medical University, Nanjing, China, 210000

4 QSPMed Technologies, Nanjing, China, 210000

5 Department of Biomedical Engineering, School of Medicine, Johns Hopkins University, Baltimore, USA, 21205

6 Key Laboratory of Cardiovascular & Cerebrovascular Medicine, Drug Target and Drug Discovery Center, School of Pharmacy, Nanjing Medical University, Nanjing, China, 210000

7 School of Basic Medical Sciences, Nanjing Medical University, Nanjing, China, 210000

--A combined PDF file including Figures S1–S11

Supplementary Fig S1. Additional quantitative model calibration of pathway signal transduction. See also Fig. 2.

Supplementary Fig S2. Additional model calibration and validation of cell signaling, hypoxia regulation, cell metabolism, and oxidative stress modules. See also Figs 3 and 4.

Supplementary Fig S3. Heatmap representation of differential biomarker response in BMEC under combined VEGF and OGD stimulation. See also Fig. 5.

Supplementary Fig S4. Heatmap representation of differential biomarker response in BMEC under combined TNFα and OGD stimulation. See also Fig. 5.

Supplementary Fig S5. The complete global sensitivity analysis results with inflammatory factor production as the output of interest. See also Fig. 6.

Supplementary Fig S6. The complete global sensitivity analysis results with growth factor production as the output of interest. See also Fig. 6.

Supplementary Fig S7. The complete global sensitivity analysis results with tight junction protein expression as the output of interest. See also Fig. 6.

Supplementary Fig S8. The complete global sensitivity analysis results with all functional biomarkers as the output of interest. See also Fig. 6.

Supplementary Fig S9. Virtual single cell modeling to analyze the effectiveness of targeting HIF1α in the regulation of BMEC functional phenotypes under OGD/R. See also Fig. 6.

Supplementary Fig S10. Virtual single cell modeling to analyze the effectiveness of targeting p53 in the regulation of BMEC functional phenotypes under OGD/R. See also Fig. 6.

Supplementary Fig S11. A detailed diagram of model structure. See also Fig. 1.

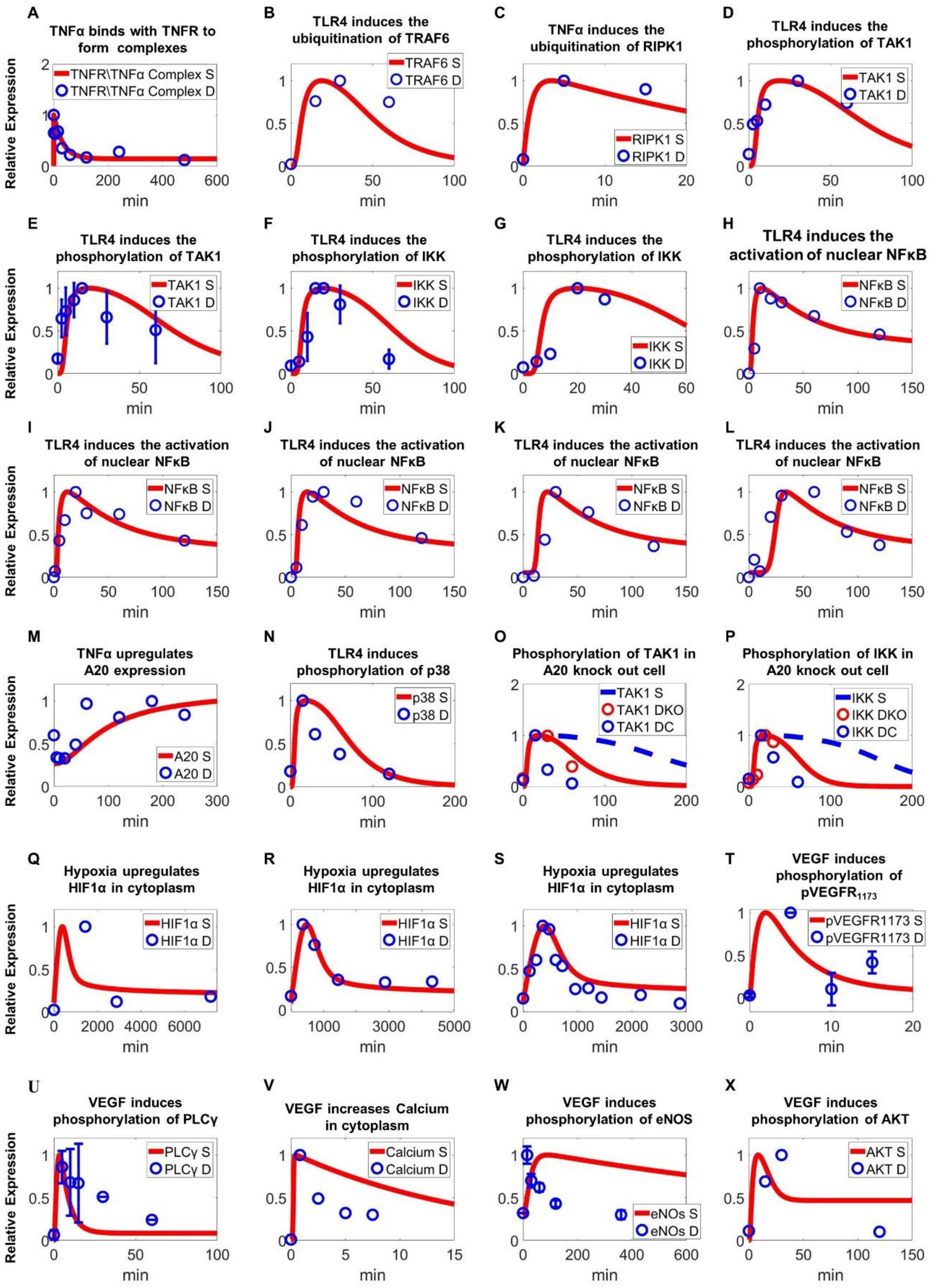

Figure S1. Additional quantitative model calibration of pathway signal transduction. (A) TNFα binds with TNFR to form signaling complexes [1]. (B) LPS (10ng/ml) induces the ubiquitination of TRAF6 [2]. (C) TNFα (20ng/ml) induces the ubiquitination of RIPK1 [3]. (D-G) LPS (10μg/ml, 1μg/ml, 100ng/ml) induces the phosphorylation of TAK1 and IKK [4, 5, 6, 7]. (H-L) LPS (5000ng/ml, 500ng/ml, 50ng/ml, 5ng/ml, 1.5ng/ml) induces the activation of NFκB [8]. (M) TNFα (10ng/ml) upregulates the expression of

A20 [9]. (N) LPS (10ng/ml) induces the phosphorylation of p38 [10]. (O-P) LPS (1ug/ml) induces the phosphorylation of TAK1 and IKK and alters the levels in A20 knock-out cells compared to normal cells. The solid line represents the untreated group, while the dashed line represents A20 knockout group [11]. (Q-S) Hypoxia (0.9%O_2, 1%O_2, 3%O_2) upregulates HIF1α in cytoplasm [12, 13, 14]. (T-X) VEGF (50ng/ml, 100ng/ml) induces the phosphorylation of PLCγ, eNOS, AKT, and increases calcium concentration in the cytoplasm [15, 16, 17, 18, 19]. (A-X) Y axes are relative expression levels (normalized to maximum). S, simulation; D, experimental data.

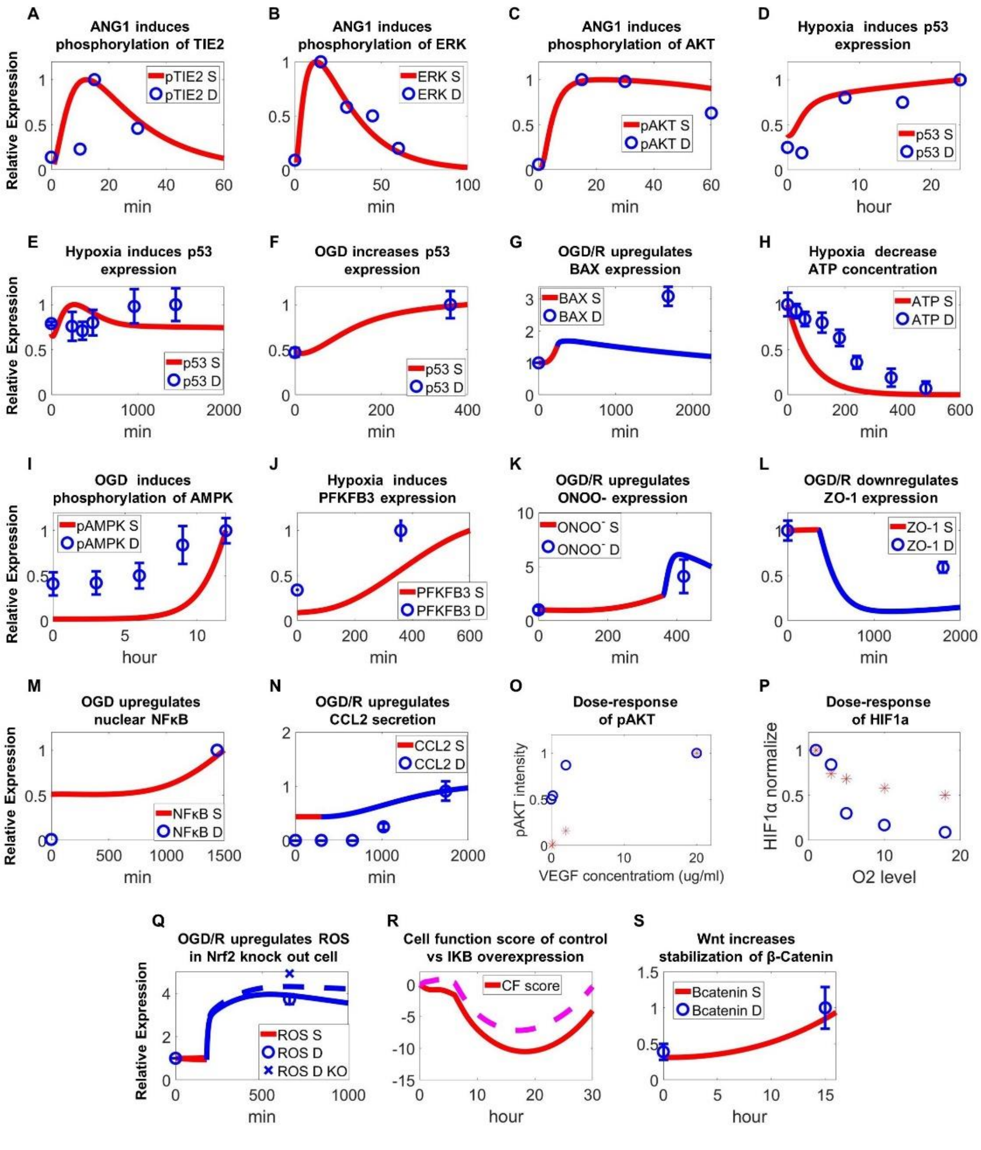

Figure S2. Additional model calibration and validation of cell signaling, hypoxia regulation, cell metabolism, and oxidative stress modules. (A-C) ANG1 (100ng/ml, 400ng/ml) induces the phosphorylation of TIE2, ERK, AKT [20, 21]. (D-G) Hypoxia (3%O_2, 0.1%O_2) and OGD induces p53 expression change, and p53 induces BAX expression under OGD/R [22, 23, 24, 25]. (H-J) Hypoxia (1%O_2,

0.1%O_2) and OGD differentially regulates ATP, AMPK activation and PFKFB3 expression [26, 27, 28]. (K-L) OGD/R induces $ONOO^-$ and represses cell ZO-1 expression [29, 30]. (M) OGD induces the activation of NFκB [31]; (N) OGD/R increase CCL2 secretion [32]. (O-P) Dose-response of AKT under different VEGF conditions and dose-response of HIF1α under different O_2 concentration [33, 34]; (Q) The alteration in ROS levels between Nrf2 knock-out cells and normal cells under conditions of OGD/R [35]. (R) Cell function under OGD/R: comparison of normal cells with cells treated to enhance IKB activity. The solid line represents the untreated group, while the dashed line represents the group with enhanced IKB activity. (S) Wnt (10ng/ml) increases stabilization of β-catenin [36]. (A-S) Y axes are relative expression levels (normalized to maximum). S, simulation; D, experimental data; CF score, Cell function score.

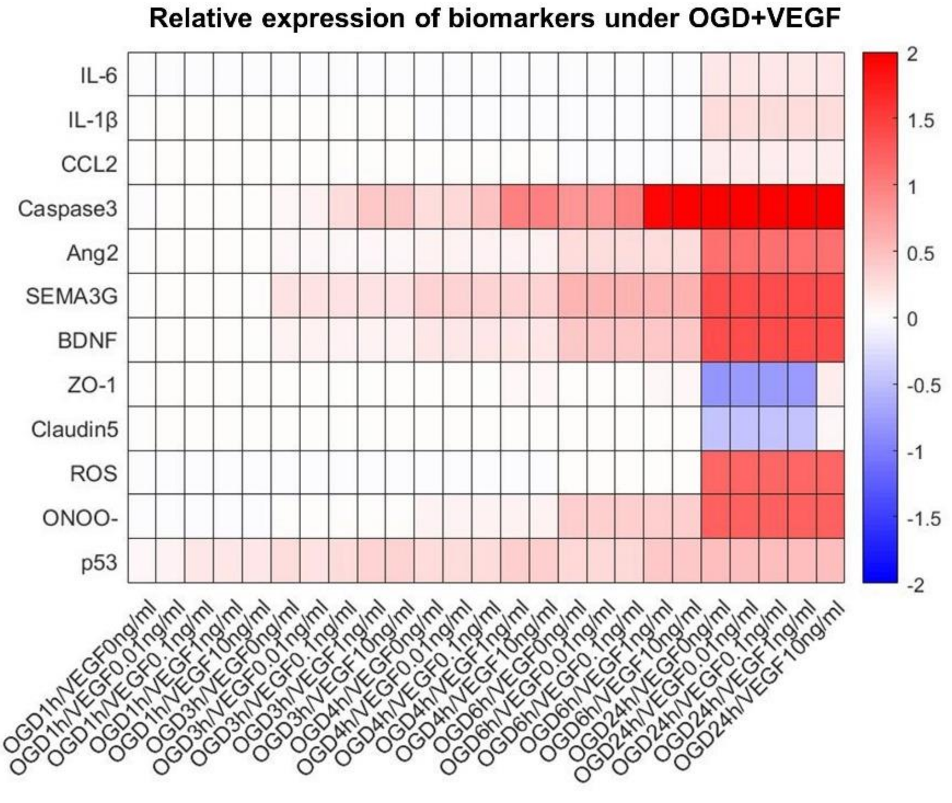

Figure S3. Heatmap representation of differential biomarker response in BMEC under combined VEGF and OGD stimulation. Under various experimental OGD conditions (with different OGD times – 1h, 3h, 4h, 6h, 24h) combined with VEGF stimulation (0 ng/ml, 0.01 ng/ml, 0.1 ng/ml, 1 ng/ml, 10 ng/ml), an expression change heatmap was generated to depict the relative dynamic changes in inflammatory cytokine secretion (IL-6, IL-1β, CCL2), growth factor secretion (Ang2, SEMA3G, BDNF), tight junction protein expression (ZO-1, Claudin5), generation of free radical species (ROS, $ONOO^-$), and expression of apoptosis markers (p53, Caspase3) in BMECs. Relative expression fold changes of all markers were computed with respect to their initial expression under control condition (normoxia and normal glucose) and displayed in red-blue color map (values were log2 transformed).

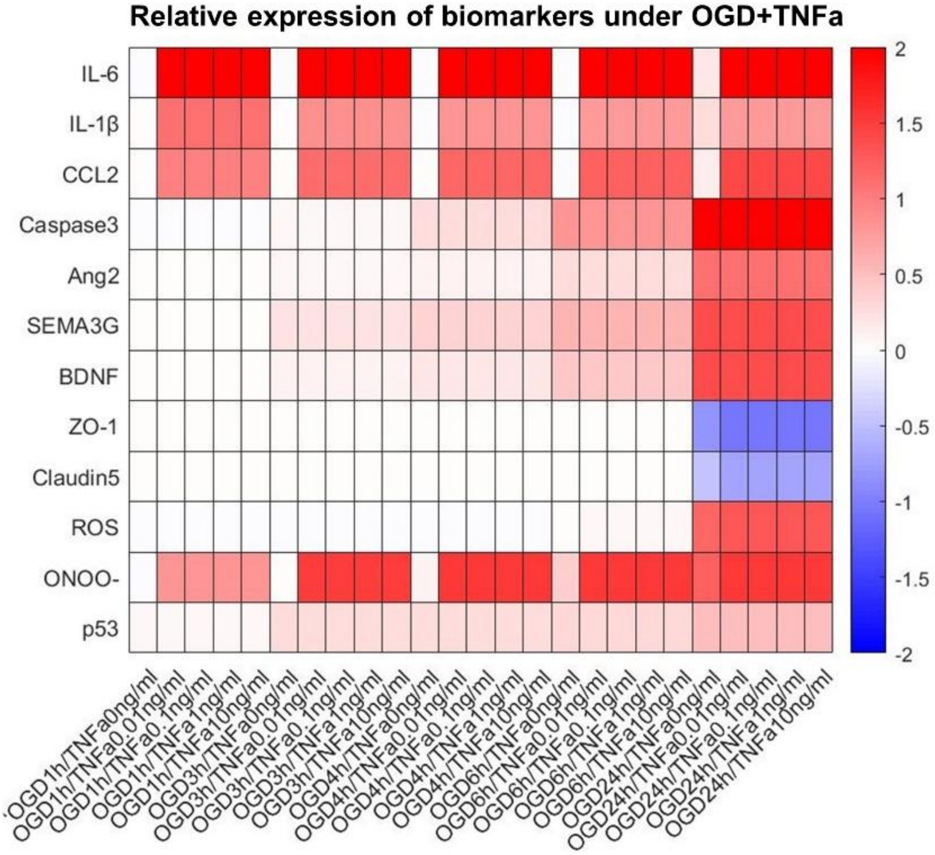

Figure S4. Heatmap representation of differential biomarker response in BMEC under combined TNFα and OGD stimulation. Relate to Figure 5. Under various experimental OGD conditions (with different OGD times – 1h, 3h, 4h, 6h, 24h) combined with TNFα stimulation (0 ng/ml, 0.01 ng/ml, 0.1 ng/ml, 1 ng/ml, 10 ng/ml), an expression change heatmap was generated to depict the relative dynamic changes in inflammatory cytokine secretion (IL-6, IL-1β, CCL2), growth factor secretion (Ang2, SEMA3G, BDNF), tight junction protein expression (ZO-1, Claudin5), generation of free radical species (ROS, ONOO-), and expression of apoptosis markers (p53, Caspase3) in BMECs. Relative expression fold changes of all markers were computed with respect to their initial expression under control condition (normoxia and normal glucose) and displayed in red-blue color map (values were log2 transformed).

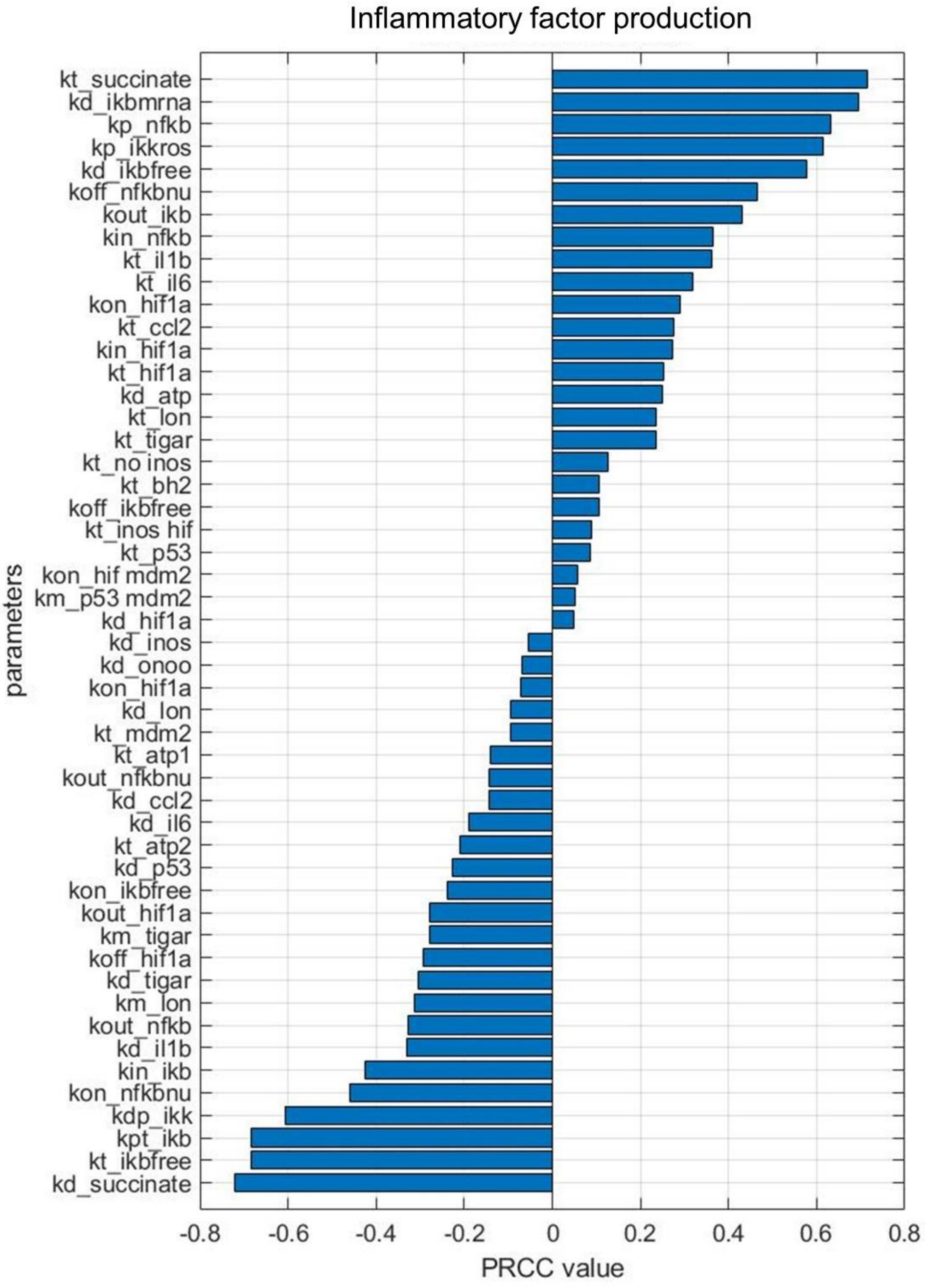

Figure S5. The complete global sensitivity analysis results with inflammation factor production as the output of interest. We utilized the PRCC algorithm to analyze the influence of diverse parameters on BMEC production of key inflammatory factors. A portion of the results have been showcased in Figure 6, with the full results presented in this figure. Parameters were ranked in terms of their absolute influences (PRCC values) on the outcome.

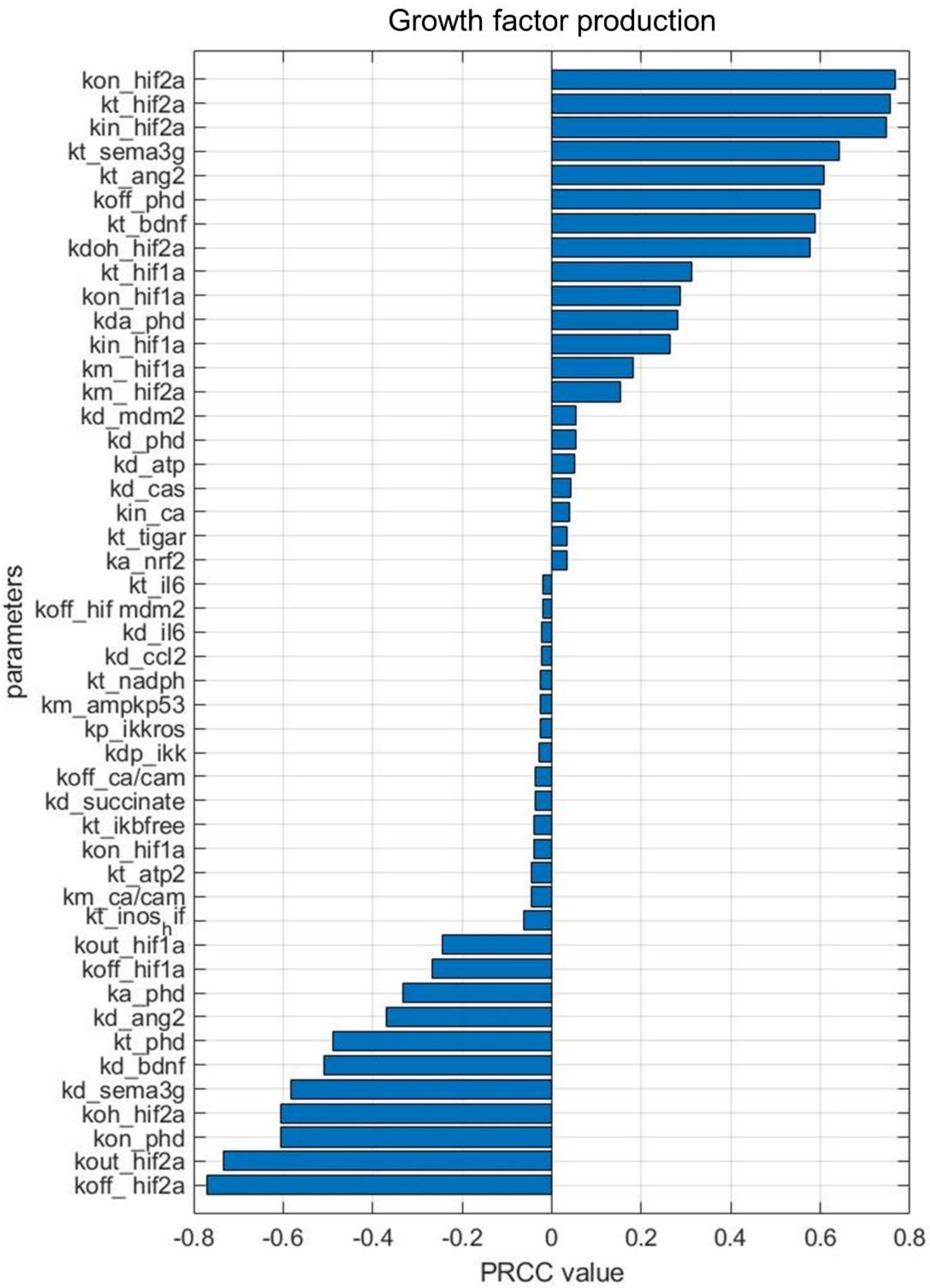

Figure S6. The complete global sensitivity analysis results with growth factor production as the output of interest. We utilized the PRCC algorithm to analyze the influence of diverse parameters on BMEC production of key growth factors. A portion of the results have been showcased in Figure 6, with the full results presented in this figure. Parameters were ranked in terms of their absolute influences (PRCC values) on the outcome.

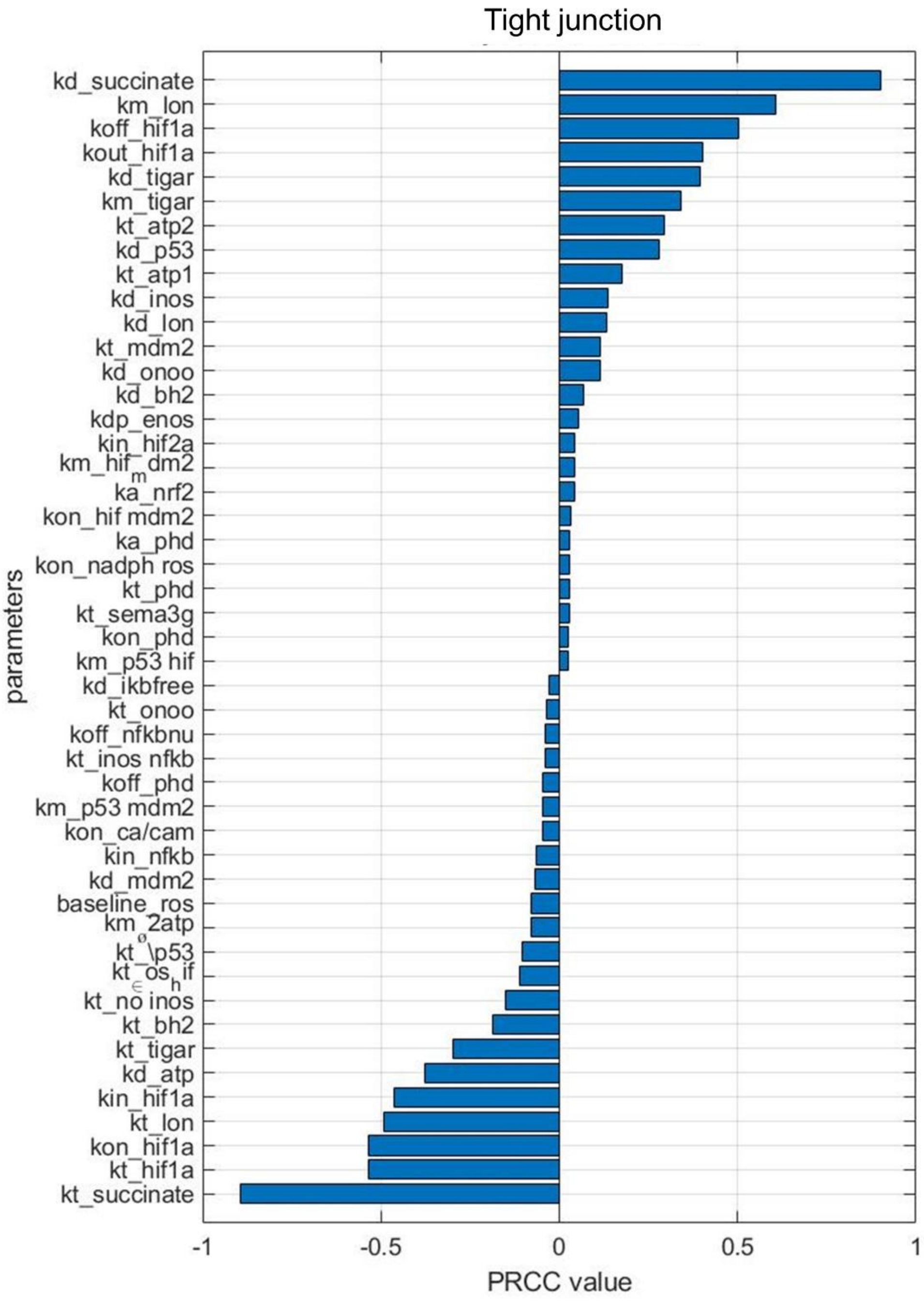

Figure S7. The complete global sensitivity analysis results with tight junction protein expression as the output of interest. We utilized the PRCC algorithm to analyze the influence of diverse parameters on BMEC expression of key tight junction proteins. A portion of the results have been showcased in Figure 6, with the full results presented in this figure. Parameters were ranked in terms of their absolute influences (PRCC values) on the outcome.

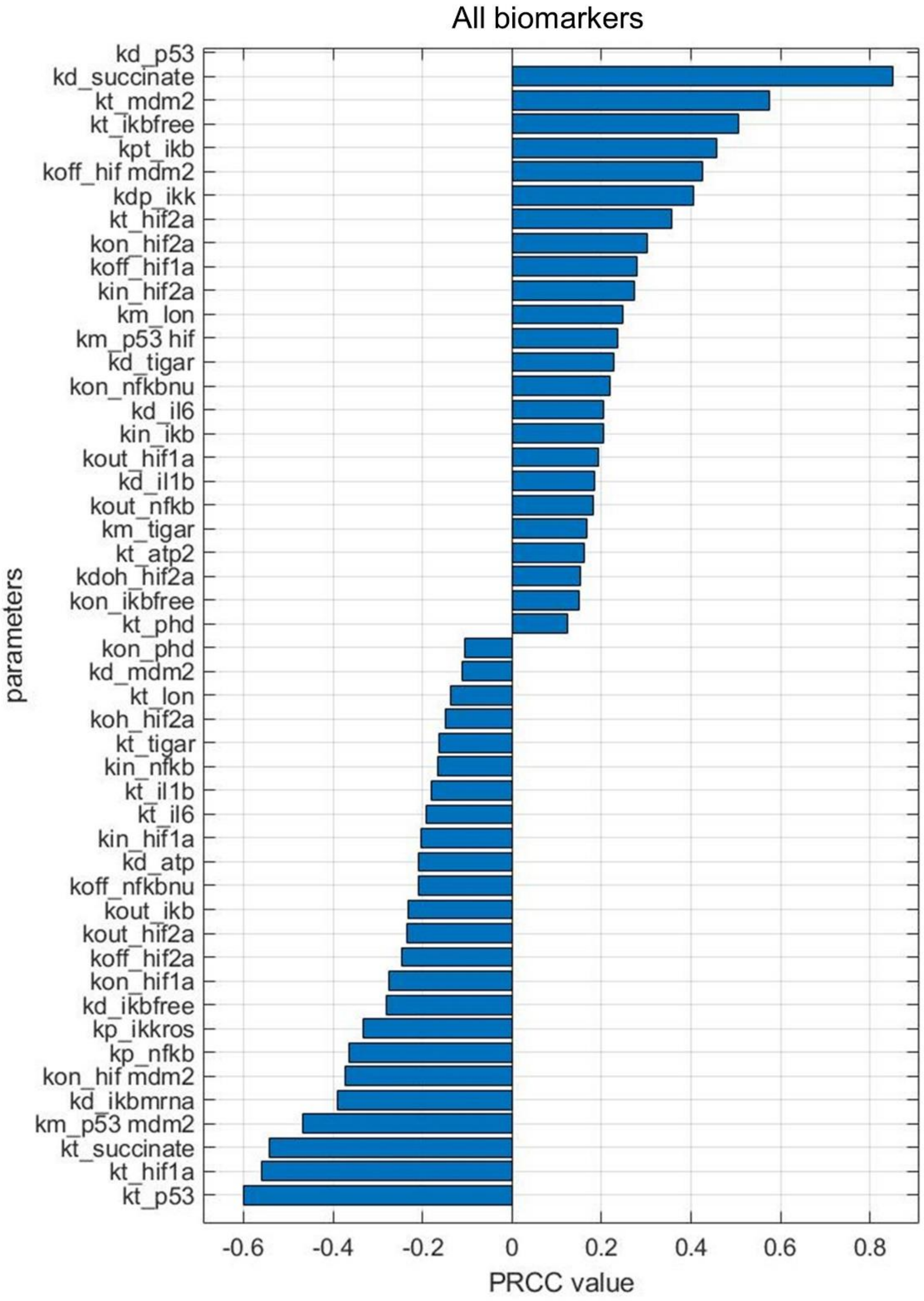

Figure S8. The complete global sensitivity analysis results with all functional biomarkers as the output of interest. We utilized the PRCC algorithm to analyze the influence of diverse parameters on the BMEC expression/secretion of all functional biomarkers. A portion of the results have been showcased in Figure 6, with the full results presented in this figure. Parameters were ranked in terms of their absolute influences (PRCC values) on the outcome.

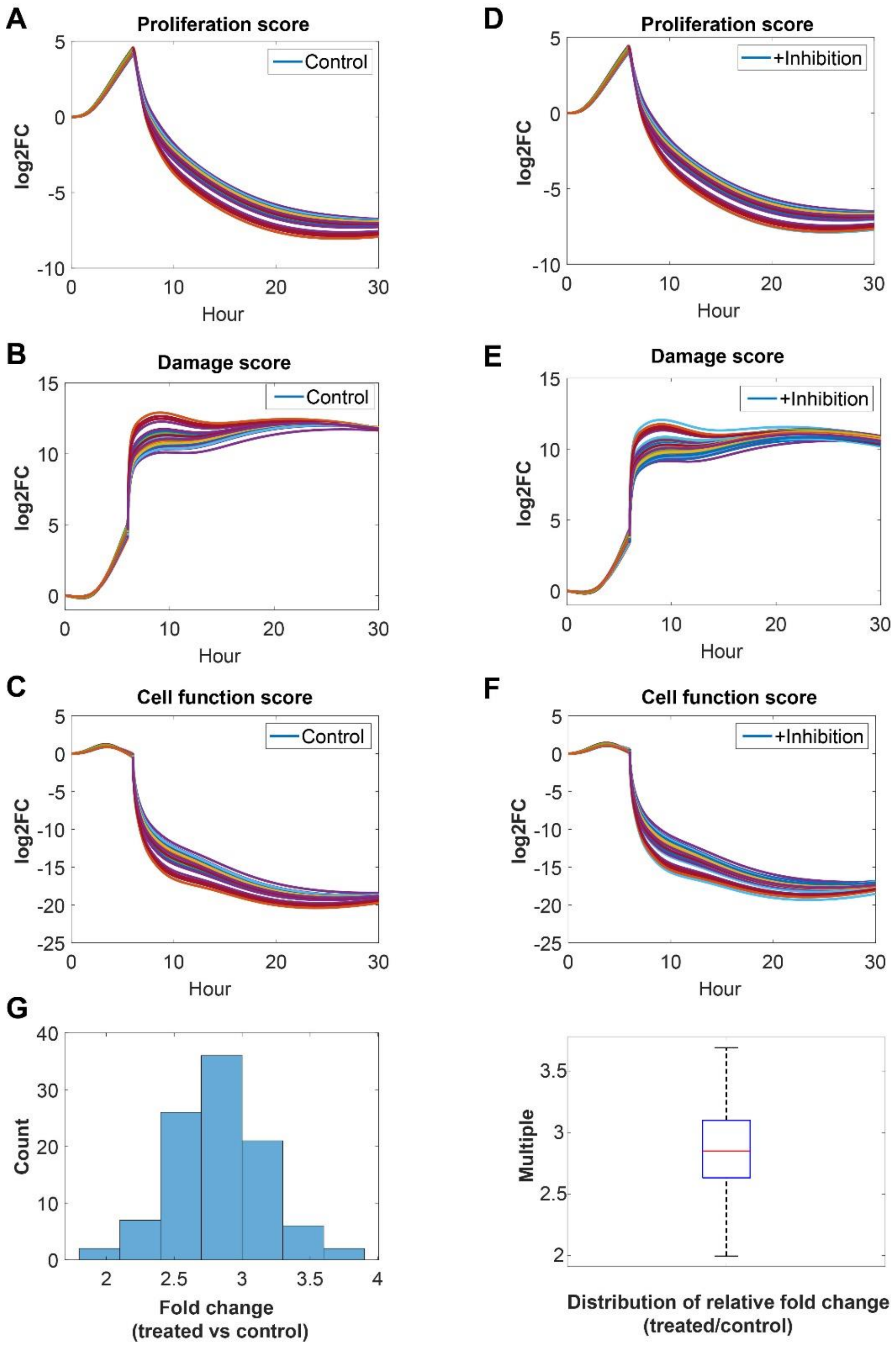

Figure S9. Virtual single cell modeling to analyze the effectiveness of targeting HIF1α in the regulation of BMEC functional phenotypes under OGD/R. We simulated a cohort of 100 virtual cells under the same OGD/R condition (OGD 6h, Reoxygenation 24h). These cells were assessed for (A) proliferation score, (B) damage score, and the (C) overall cell function score over time. (D-F) Additionally, inhibition of HIF1α was simulated in the virtual cells and the individual BMEC proliferation/damage/cell function scores were computed and displayed. (G) Under OGD/R, the relative fold changes of the overall cell function scores of the BMEC cohort with HIF1α inhibition compared to scores of control BMECs were computed and displayed in histograms and boxplots, which indicated moderate upregulation toward the direction of BMEC protection.

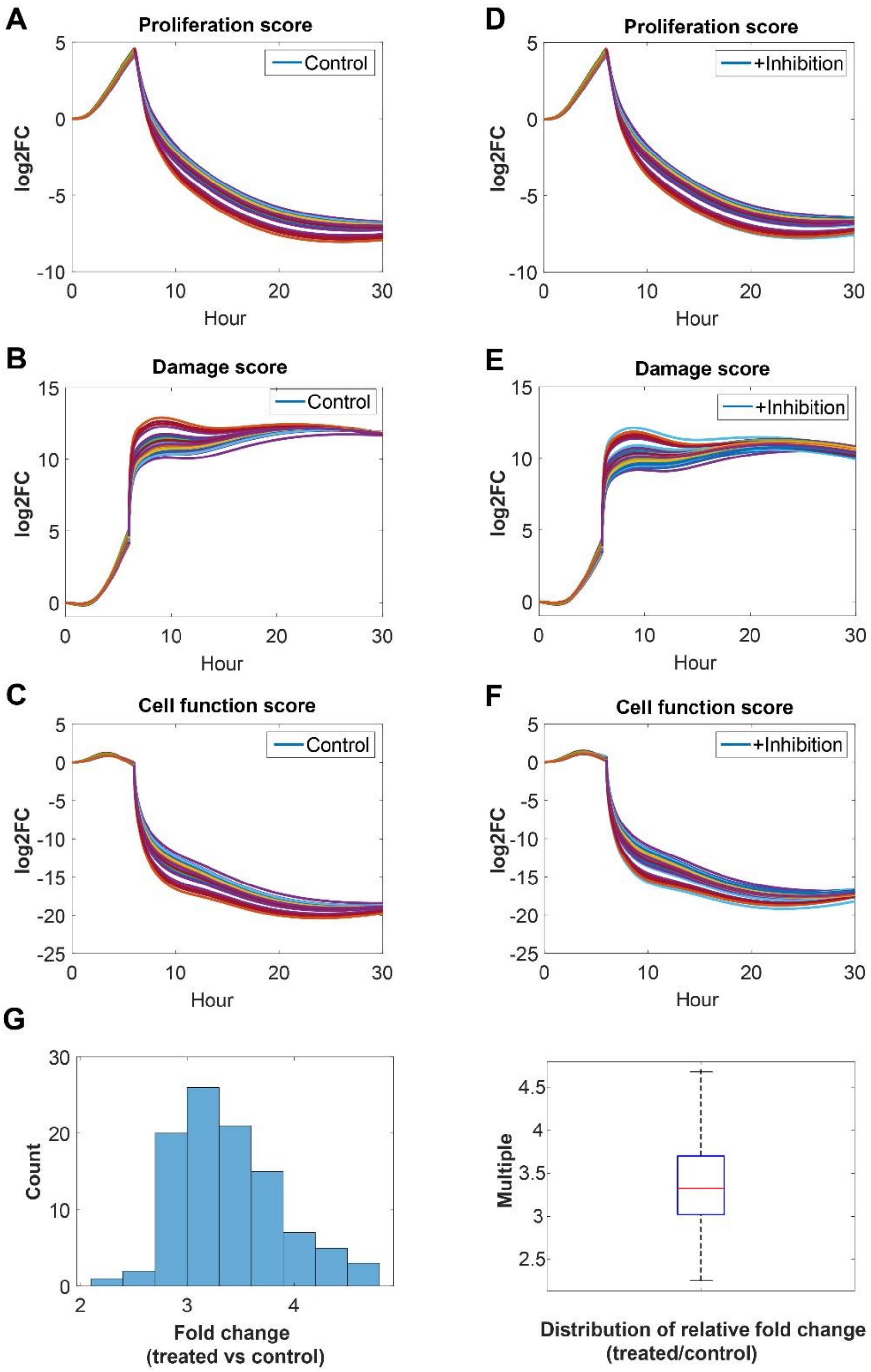

Figure S10. Virtual single cell modeling to analyze the effectiveness of targeting p53 in the regulation of BMEC functional phenotypes under OGD/R. We simulated a cohort of 100 virtual cells under the same OGD/R condition (OGD 6h, Reoxygenation 24h). These cells were assessed for (A) proliferation score, (B) damage score, and the (C) overall cell function score over time. (D-F) Additionally, inhibition of p53 was simulated in the virtual cells and the individual BMEC proliferation/damage/cell function scores were computed and displayed. (G) Under OGD/R, the relative fold changes of the overall cell function scores of the BMEC cohort with p53 inhibition compared to scores of control BMECs were computed and displayed in histograms and boxplots, which indicated again moderate upregulation toward the direction of BMEC protection.

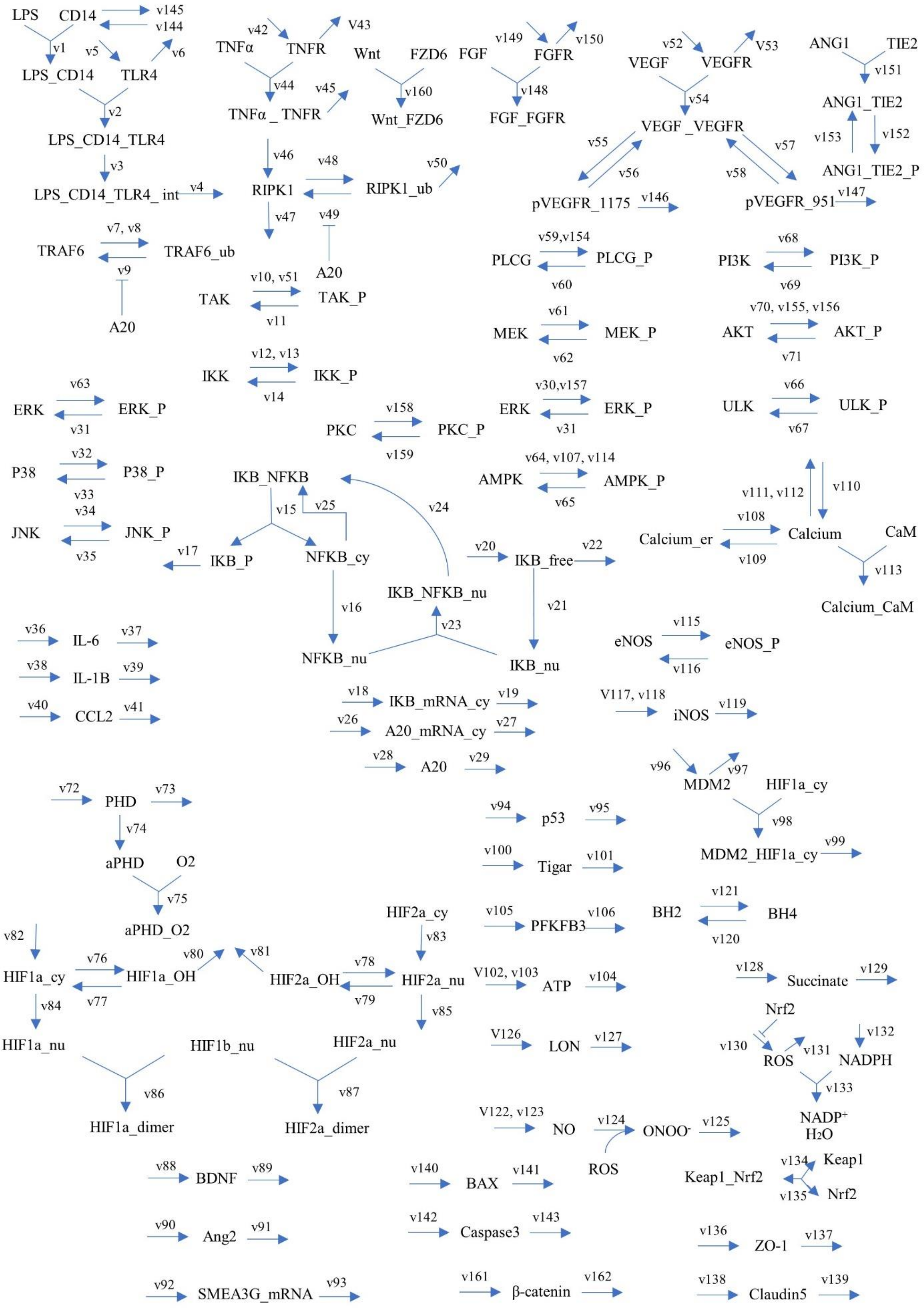

Figure S11. A detailed diagram of model structure. All unique model species and reaction fluxes were shown with labels that can be referred to in Supplemental Tables S1.

References for Supplementary Materials

1. Micheau, O. and J. Tschopp, *Induction of TNF receptor I-mediated apoptosis via two sequential signaling complexes.* Cell, 2003. **114**(2): p. 181-90.
2. Xiong, Y., et al., *Endotoxin tolerance impairs IL-1 receptor-associated kinase (IRAK) 4 and TGF-beta-activated kinase 1 activation, K63-linked polyubiquitination and assembly of IRAK1, TNF receptor-associated factor 6, and IkappaB kinase gamma and increases A20 expression.* J Biol Chem, 2011. **286**(10): p. 7905-7916.
3. Tang, Y., et al., *K63-linked ubiquitination regulates RIPK1 kinase activity to prevent cell death during embryogenesis and inflammation.* Nat Commun, 2019. **10**(1): p. 4157.
4. Wang, J.S., et al., *TAK1 inhibition-induced RIP1-dependent apoptosis in murine macrophages relies on constitutive TNF-alpha signaling and ROS production.* J Biomed Sci, 2015. **22**(1): p. 76.
5. Ouyang, C., et al., *Transforming growth factor (TGF)-beta-activated kinase 1 (TAK1) activation requires phosphorylation of serine 412 by protein kinase A catalytic subunit alpha (PKACalpha) and X-linked protein kinase (PRKX).* J Biol Chem, 2014. **289**(35): p. 24226-37.
6. Villacorta, L., et al., *Electrophilic nitro-fatty acids inhibit vascular inflammation by disrupting LPS-dependent TLR4 signalling in lipid rafts.* Cardiovasc Res, 2013. **98**(1): p. 116-24.
7. Pinte, S., et al., *Endothelial Cell Activation Is Regulated by Epidermal Growth Factor-like Domain 7 (Egfl7) during Inflammation.* J Biol Chem, 2016. **291**(46): p. 24017-24028.
8. Bagaev, A.V., et al., *Elevated pre-activation basal level of nuclear NF-kappaB in native macrophages accelerates LPS-induced translocation of cytosolic NF-kappaB into the cell nucleus.* Sci Rep, 2019. **9**(1): p. 4563.
9. Pinna, F., et al., *A20/TNFAIP3 Discriminates Tumor Necrosis Factor (TNF)-Induced NF-kappaB from JNK Pathway Activation in Hepatocytes.* Front Physiol, 2017. **8**: p. 610.
10. Khatchadourian, A., et al., *Dynamics and regulation of lipid droplet formation in lipopolysaccharide (LPS)-stimulated microglia.* Biochim Biophys Acta, 2012. **1821**(4): p. 607-17.
11. Soni, D., et al., *Deubiquitinase function of A20 maintains and repairs endothelial barrier after lung vascular injury.* Cell Death Discovery, 2018. **4**(1).
12. Jiang, Y.-Z., et al., *Distinct roles of HIF1A in endothelial adaptations to physiological and ambient oxygen.* Molecular and Cellular Endocrinology, 2014. **391**(1-2): p. 60-67.
13. Liang, X., et al., *Hypoxia Enhances Endothelial Intercellular Adhesion Molecule 1 Protein Level Through Upregulation of Arginase Type II and Mitochondrial Oxidative Stress.* Front Physiol, 2019. **10**: p. 1003.
14. Bartoszewski, R., et al., *Primary endothelial cell-specific regulation of hypoxia-inducible factor (HIF)-1 and HIF-2 and their target gene expression profiles during hypoxia.* FASEB J, 2019. **33**(7): p. 7929-7941.
15. Li, J., et al., *Orai1 and CRAC Channel Dependence of VEGF-Activated Ca 2+ Entry and Endothelial Tube Formation.* Circulation Research, 2011. **108**(10): p. 1190-1198.
16. Watari, K., et al., *NDRG1 activates VEGF-A-induced angiogenesis through PLCgamma1/ERK signaling in mouse vascular endothelial cells.* Commun Biol, 2020. **3**(1): p. 107.
17. Basagiannis, D., et al., *Dynasore impairs VEGFR2 signalling in an endocytosis-independent manner.* Scientific Reports, 2017. **7**(1).
18. Radisavljevic, Z., H. Avraham, and S. Avraham, *Vascular Endothelial Growth Factor Up-regulates ICAM-1 Expression via the Phosphatidylinositol 3 OH-kinase/AKT/Nitric Oxide Pathway and Modulates Migration of Brain Microvascular Endothelial Cells.* Journal of Biological Chemistry, 2000. **275**(27): p. 20770-20774.
19. Chen, M., et al., *Pim1 kinase promotes angiogenesis through phosphorylation of endothelial nitric oxide synthase at Ser-633.* Cardiovascular Research, 2016. **109**(1): p. 141-150.
20. Siddiqui, M.R., et al., *Angiopoietin-1 Regulates Brain Endothelial Permeability through PTPN-2 Mediated Tyrosine Dephosphorylation of Occludin.* PLoS One, 2015. **10**(6): p. e0130857.
21. Fukuhara, S., et al., *Differential function of Tie2 at cell-cell contacts and cell-substratum contacts*

regulated by angiopoietin-1. Nat Cell Biol, 2008. **10**(5): p. 513-26.

22. Wang, Z., et al., *Divergent changes of p53 in pulmonary arterial endothelial and smooth muscle cells involved in the development of pulmonary hypertension.* Am J Physiol Lung Cell Mol Physiol, 2019. **316**(1): p. L216-L228.
23. Li, J., et al., *p53 participates in the protective effects of ischemic post-conditioning against OGD-reperfusion injury in primary cultured spinal cord neurons.* Neurosci Lett, 2017. **638**: p. 129-134.
24. Liu, D., et al., *Circ_0000566 contributes oxygen-glucose deprivation and reoxygenation (OGD/R)-induced human brain microvascular endothelial cell injury via regulating miR-18a-5p/ACVR2B axis.* Metab Brain Dis, 2023. **38**(4): p. 1273-1284.
25. Leszczynska, K.B., et al., *Hypoxia-induced p53 modulates both apoptosis and radiosensitivity via AKT.* J Clin Invest, 2015. **125**(6): p. 2385-98.
26. Cao, X., et al., *Persistent oxygen-glucose deprivation induces astrocytic death through two different pathways and calpain-mediated proteolysis of cytoskeletal proteins during astrocytic oncosis.* Neurosci Lett, 2010. **479**(2): p. 118-22.
27. Cao, Y., et al., *PFKFB3-mediated endothelial glycolysis promotes pulmonary hypertension.* Proc Natl Acad Sci U S A, 2019. **116**(27): p. 13394-13403.
28. Natarajan, V., et al., *Oxygen Glucose Deprivation Induced Prosurvival Autophagy Is Insufficient to Rescue Endothelial Function.* Front Physiol, 2020. **11**: p. 533683.
29. Cavdar, Z., et al., *Resveratrol reduces matrix metalloproteinase-2 activity induced by oxygen-glucose deprivation and reoxygenation in human cerebral microvascular endothelial cells.* Int J Vitam Nutr Res, 2012. **82**(4): p. 267-74.
30. Liu, J., et al., *The Protective Effects of Juglanin in Cerebral Ischemia Reduce Blood-Brain Barrier Permeability via Inhibition of VEGF/VEGFR2 Signaling.* Drug Des Devel Ther, 2020. **14**: p. 3165-3175.
31. Castri, P., et al., *Poly(ADP-ribose) polymerase-1 and its cleavage products differentially modulate cellular protection through NF-kappaB-dependent signaling.* Biochim Biophys Acta, 2014. **1843**(3): p. 640-51.
32. Dimitrijevic, O.B., et al., *Effects of the chemokine CCL2 on blood-brain barrier permeability during ischemia-reperfusion injury.* J Cereb Blood Flow Metab, 2006. **26**(6): p. 797-810.
33. Matsushita, K., et al., *Vascular endothelial growth factor regulation of Weibel-Palade-body exocytosis.* Blood, 2005. **105**(1): p. 207-14.
34. Bracken, C.P., et al., *Cell-specific regulation of hypoxia-inducible factor (HIF)-1alpha and HIF-2alpha stabilization and transactivation in a graded oxygen environment.* J Biol Chem, 2006. **281**(32): p. 22575-85.
35. Xu, X., et al., *Nrf2/ARE pathway inhibits ROS-induced NLRP3 inflammasome activation in BV2 cells after cerebral ischemia reperfusion.* Inflamm Res, 2018. **67**(1): p. 57-65.
36. Laksitorini, M.D., et al., *Modulation of Wnt/β-catenin signaling promotes blood-brain barrier phenotype in cultured brain endothelial cells.* Sci Rep, 2019. **9**(1): p. 19718.
